# Glycosphingolipid-Glycan Signatures of Acute Myeloid Leukemia Cell Lines Reflect Hematopoietic Differentiation

**DOI:** 10.1021/acs.jproteome.1c00911

**Published:** 2022-02-16

**Authors:** Di Wang, Tao Zhang, Katarina Madunić, Antonius A. de Waard, Constantin Blöchl, Oleg A. Mayboroda, Marieke Griffioen, Robbert M. Spaapen, Christian G. Huber, Guinevere S.M. Lageveen-Kammeijer, Manfred Wuhrer

**Affiliations:** † Center for Proteomics and Metabolomics, Leiden University Medical Center, Postbus 9600, 2300 RC Leiden, The Netherlands; ‡ Department of Hematology, Leiden University Medical Center, Postbus 9600, 2300 RC Leiden, The Netherlands; § Department of Immunopathology, Sanquin Research, 1066 CX Amsterdam, The Netherlands; ∥ Landsteiner Laboratory, Amsterdam UMC, University of Amsterdam, 1066 CX Amsterdam, The Netherlands; ⊥ Department of Biosciences, University of Salzburg, Hellbrunnerstrasse 34, 5020 Salzburg, Austria

**Keywords:** glycosphingolipids, acute myeloid leukemia, glycosyltransferases, hematopoietic transcription factors, mass spectrometry, porous graphitized carbon liquid chromatography

## Abstract

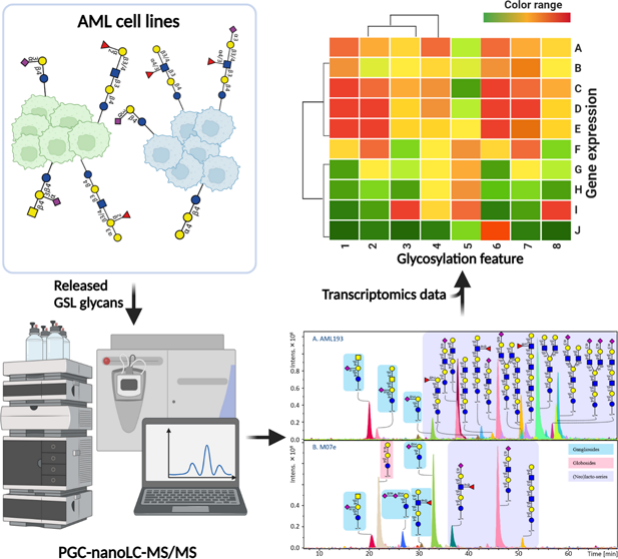

Aberrant expression of certain glycosphingolipids (GSLs) is associated with the differentiation of acute myeloid leukemia (AML) cells. However, the expression patterns of GSLs in AML are still poorly explored because of their complexity, the presence of multiple isomeric structures, and tedious analytical procedures. In this study, we performed an in-depth GSL glycan analysis of 19 AML cell lines using porous graphitized carbon liquid chromatography-mass spectrometry revealing strikingly different GSL glycan profiles between the various AML cell lines. The cell lines of the M6 subtype showed a high expression of gangliosides with α2,3-sialylation and Neu5Gc, while the M2 and M5 subtypes were characterized by high expression of (neo)lacto-series glycans and Lewis A/X antigens. Integrated analysis of glycomics and available transcriptomics data revealed the association of GSL glycan abundances with the transcriptomics expression of certain glycosyltransferases (GTs) and transcription factors (TFs). In addition, correlations were found between specific GTs and TFs. Our data reveal TFs *GATA2*, *GATA1*, and *RUNX1* as candidate inducers of the expression of gangliosides and sialylation via regulation of the GTs *ST3GAL2* and *ST8SIA1*. In conclusion, we show that GSL glycan expression levels are associated with hematopoietic AML classifications and TF and GT gene expression. Further research is needed to dissect the regulation of GSL expression and its role in hematopoiesis and associated malignancies.

## Introduction

Glycosphingolipids (GSLs) are amphipathic compounds consisting of a hydrophobic ceramide backbone which is covalently linked to a hydrophilic carbohydrate residue. In mammals, GSLs can be classified into three subgroups on the basis of common neutral core structures, namely, gangliosides, (neo)lacto-series, and globosides.^1^ The GSL glycans are complex because of the diversity in monosaccharides, the order of monosaccharides, anomeric configuration, branching, and linkage positions.^2^ Unlike other biopolymers, the biosynthesis of GSLs is not template-driven but rather depends on the combined action of the enzymes involved in the biosynthesis.^1,3^ Moreover, GSL biosynthesis is known to be transcriptionally regulated at the glycosyltransferase (GT) level.^4^ A previous study investigated the *N*- and *O*-glycome of AML cell lines and explored the potential roles of relevant GTs and transcription factors (TFs) in the regulation of the glycan phenotype of the studied acute myeloid leukemia (AML) cell lines.^5^ However, still little is known about the gene network that coordinates the expression of GSL-synthesizing enzymes.^1,6^

Alterations of glycosylation including changes of sialylation (α2,3/6/8-sialylation) and fucosylation (blood H antigens, Lewis antigens) have been described for various hematological malignancies for both glycoproteins and GSLs.^7^ GSLs play essential roles in cellular processes including adhesion, proliferation, differentiation, and recognition.^1,6,8^ Abnormal GSL expression is associated with the development of many types of cancers including leukemia.^9^ A subtype of leukemia, AML, is a heterogeneous clonal disorder of hemopoietic progenitors of the myeloid lineage which gives rise to red blood cells (RBCs), several different white blood cells, and platelets.^10^ AML is the second most common type of leukemia diagnosed in adults and children, with most cases occurring in adults. The mortality rate of AML in the United States is estimated at 11,400 for 2021, accounting for approximately 48% of all leukemia cases.^11^ AML can be classified into eight subtypes (M0–M7), according to the French–American–British (FAB) classification system which was established in 1976.^12^ The subtypes are defined based on morphological and cytochemical characteristics of the leukemia cells. Basically, subtypes M0 to M5 are all derived from certain immature white blood cells called myeloblasts, M6 AML cells are initiated from an immature form of RBCs (erythrocytes), and M7 AML cells are from immature forms of cells which make platelets (megakaryocytes).

It is suspected that GSLs, which are present on the cell surface, might participate in myelopoiesis.^13^ Previous studies have indicated that certain GSLs play a critical role in the differentiation of AML cells.^13^ For example, the incorporation of GSL GM3 (NeuAcα2-3Galβ1-4Glcβ1-Cer) into HL60 cells resulted in the growth inhibition and monocytic differentiation of the cells.^14^ During the differentiation of monocytic THP-1 cells into macrophages, α2,6-sialylation slightly decreased and GM3 expression increased which correlated directly with the overexpression of ST3GAL5.^15^ The concerted upregulation of ST3GAL5 and GM3 resulted from the activation of the PKC/ERK pathway during the differentiation of HL60 cells.^16^ In addition, the decrease of GSL Lc3 (GlcNAcβ1-3Galβ1-4Glcβ1-Cer) expression in HL60 and NB4 was induced by treatment with either all-trans retinoic acid which can stimulate the differentiation of cells into the neutrophil lineage or phorbol 12-myristate 13-acetate which induced the differentiation of cells along the monocyte lineage.

In this study, we characterized the expression of GSL glycans in 19 different AML cell lines belonging to different FAB subtypes. The GSL glycans were profiled, identified, and quantified using porous graphitized carbon nanoliquid chromatography hyphenated with mass spectrometry via electrospray ionization (PGC-nanoLC–ESI-MS/MS). Overall, a striking diversity of GSL glycan expression was found between cell lines. In addition, the association of GSL glycan expression with GT and TF gene expression and with AML classification was explored.

## Materials and Methods

### Materials

Chloroform and trifluoroacetic acid (TFA) were purchased from Merck (Darmstadt, Germany). Methanol (MeOH) of ultra LC–MS grade was obtained from Actuall Chemical (Randmeer, The Netherlands). NaBH_4_, Dowex cation-exchange resin (50 W-X8), HCl, and ammonium bicarbonate were purchased from Sigma-Aldrich (St. Louis, MO). KOH and 100% glacial acetic acid were from Honeywell Fluka (Charlotte, NC). A Sep-Pak tC18 reverse-phase (RP) solid-phase extraction (SPE) cartridge (50 mg) was obtained from Waters (Milford, MA). Ziptip C18 was obtained from Millipore (Amsterdam, The Netherlands), and SPE bulk sorbent Carbograph was purchased from Grace Discovery Sciences (Columbia, TN). TopTip (microspin column; empty column) was obtained from Glygen Corporation (Columbia, MD), and 2-propanol was purchased from Biosolve Chemie (Dieuze, France). Acetonitrile (MeCN) of LC–MS grade was purchased from Biosolve (Valkenswaard, The Netherlands). Endoglycoceramidase I (EGCase I), 1× EGCase I reaction buffer, α2–3 neuraminidase S, α1–3,4 fucosidase, α1–2,4,6 fucosidase O, purified bovine serum albumin (BSA), and 10× GlycoBuffer 1 were purchased from New England Biolabs (Ipswich, MA). Iscove’s modified Dulbecco’s medium (IMDM) was purchased from Gibco (Thermo Fisher Scientific, Bleiswijk, The Netherlands). PenStrep was obtained from Invitrogen (Thermo Fisher Scientific). Fetal calf serum (FCS) was purchased from Bodinco (Alkmaar, the Netherlands). The granulocyte-macrophage colony-stimulating factor (GM-CSF) was obtained from Cellgenix (Freiburg, Germany). Insulin and transferrin were purchased from Sigma (Zwijndrecht, The Netherlands). Ultrapure water was generated using a Q-Gard 2 system and used for all preparations and washing steps.

### Cell Lines and Cell Culture

AML cell lines were grown at the Department of Immunopathology of Sanquin Research (Amsterdam, The Netherlands). Briefly, all cells were cultured in IMDM supplemented with heat-inactivated FCS (5% for AML193, 20% for Kasumi1 and ME-1, 10% for the others) and 1% PenStrep at 37 °C and 5% CO_2_. The AML193 medium was supplemented with 5 μg/mL insulin, 5 μg/mL transferrin, and 5 μg/mL GM-CSF, and the media of cell lines M07e and TF1 were supplemented with 20 ng/mL GM-CSF. For more information regarding each cell line, see Supplementary Information, Table S-1.

### Extraction and Purification of GSLs from AML Cell Lines

The cells were processed as described in a previous study.^17^ Briefly, prior to cell lysis, two million cells were suspended in 200 μL of H_2_O and vortexed followed by a 30 min sonification at room temperature (RT) in a glass vial. Next, 550 μL of chloroform and 350 μL of MeOH were added to the suspension. The sample was again briefly vortexed, sonicated for 30 min, and incubated for 4 h by horizontally shaking at 1000 rpm. After incubation, the sample was centrifuged for 15 min at 3000 × *g* at 20 °C. The upper phase (containing GSLs) was collected, and sequentially 400 μL of chloroform/MeOH (2:1) and 400 μL of MeOH/ H_2_O (1:1) were added to the sample. After overnight incubation with shaking at RT, two additional extraction steps were performed by collecting the upper phase (centrifugation at 3000 × *g* at 20 °C for 15 min) and adding 400 μL of MeOH/ H_2_O (1:1) to the sample followed by sonication for 10 min before centrifugation. The collected upper phases (four in total) were combined into a single vial and evaporated to dryness with vacuum. The tC18 RP-SPE cartridge was preconditioned by the sequential passing of 1 mL of chloroform/MeOH (2:1), 1 mL of MeOH, and 2 mL of MeOH/H_2_O (1:1) over the cartridge. The extracted GSLs were resuspended in 200 μL using a mixture of MeOH and H_2_O (1:1) and loaded on the tC18 RP-SPE cartridge by passing the sample three times over the cartridge. The loaded cartridge was washed with 2 mL of MeOH/H_2_O (1:1) followed by a sequential elution of the GSLs using 2 mL of MeOH and 2 mL of chloroform/MeOH (2:1). The samples were dried under vacuum in an Eppendorf Concentrator at 30 °C.

### Enzymatic Release of GSL Glycans and Purification

EGCase I from *Rhodococcus triatomea* recombinantly produced in *Escherichia coli* was used to release the glycans from the extracted GSL samples according to the manufacturer’s instruction with some slight modifications. Briefly, 36 μL of H_2_O, 4 μL of EGCase I reaction buffer, and 2 μL of EGCase I enzyme were added to each lyophilized sample. The final mixture (42 μL) was incubated at 37 °C for 36 h. After incubation, the GSL glycans were retrieved using a tC18 RP-SPE cartridge, which was preconditioned with 2 mL of MeOH and 2 mL of H_2_O prior to applying the sample onto the cartridge. The purified GSL glycans were eluted using 500 μL of H_2_O. The mixture, consisting of the elution and flowthrough, was lyophilized under vacuum.

### Reduction and Desalting of Released GSL Glycans

Reduction and desalting of the purified GSL glycans were performed as described in a previous study.^18^ Briefly, GSL glycans were reduced by adding 40 μL of 1 M NaBH_4_ in 50 mM KOH to each sample followed by incubation at 50 °C for 3 h. The reduction mixture was quenched with 4 μL of glacial acetic acid. The RP C18 Ziptips containing 50 W-X8 resin were preconditioned by applying sequentially 3 × 60 μL of 1 M HCl, 3 × 60 μL of MeOH, and 3 × 60 μL of H_2_O onto the column. Subsequently, the solution, containing glycan alditols, was loaded, and the column was washed with 100 μL of H_2_O. The flowthrough and washes were collected and dried under vacuum. To remove residual borate, 150 μL of MeOH was added twice to the samples during the drying step.

### Exoglycosidase Digestion

A pool of glycan alditols released from cell lines Molm13, HL60, ML1, ME1, HEL, PLB985, EOL-1, and AML 193 was used for exoglycosidase treatment. In brief, 5 μL of the pooled sample was added to 2 μL of 10× glycobuffer 1 and 13 μL of H_2_O followed by the addition of either 2 μL of α2–3 neuraminidase S from Streptococcus pneumoniae recombinantly expressed in *E. coli* or 4 μL of α1–2,4,6 fucosidase O from Omnitrophica *bacterium* recombinantly expressed in *E. coli*. For α1–3,4 fucosidase (from the sweet almond Prunus dulcis, recombinantly expressed in Pichia pastoris) treatment, 5 μL of the pooled sample, 11 μL of H_2_O, and 2 μL of 10× glycobuffer 1 were mixed followed by addition of 2 μL of 10× BSA and 2 μL of α1–3,4 fucosidase. Combined enzymatic digestion was performed by adding 2 μL of α2–3 neuraminidase S and 4 μL of α1–2,4,6 fucosidase O or 2 μL of α1–3,4 fucosidase to 6 μL of the pooled sample with the addition of 2 μL of 10× glycobuffer 1 and 13 μL of H_2_O. As a control, the enzyme was replaced with 10× glycobuffer 1. Each enzymatic digestion was performed twice, and all samples were incubated overnight at 37 °C followed by RP-SPE and drying of the purified glycans by vacuum centrifugation.

### Porous Graphitized Carbon Clean-up and Measurement of GSL Glycans

Lyophilized GSL glycans were reconstituted in 40 μL of H_2_O with 0.1% TFA (*v*/*v*) followed by a shaking step for 30 min. PGC SPE clean-up was performed as described previously^19^ with some slight modifications. Briefly, columns were packed with 50 μL of PGC resin slurry (containing approximately 25 μg of PGC material) followed by a conditioning step by loading 3 × 60 μL of 80% MeCN with 0.1% TFA (*v*/*v*) followed by 3 × 60 μL of H_2_O with 0.1% TFA (*v*/*v*). After sample loading, the columns were washed with 2 × 60 μL of H_2_O with 0.1% TFA (*v*/*v*). Subsequently, the GSL glycans were eluted by adding 2 × 40 μL of 60% MeCN with 0.1% TFA (*v*/*v*). The two elution fractions were combined and dried under vacuum.

Prior to MS analysis, the purified GSL glycan alditols were resuspended in 20 μL H_2_O. A volume of 1 μL of the sample was loaded onto a Dionex Ultimate 3000 nanoLC system equipped with a Hypercarb PGC-trap column (5 μm Hypercarb Kappa, 320 μm × 30 mm, home-made) and a Hypercarb PGC nanocolumn (3 μm Hypercarb Kappa, 75 μm × 100 mm, home-made). The separation platform was coupled to an amaZon speed ion trap MS (Bruker Daltonics, Bremen, Germany). Buffer A consisted of 10 mM ammonium bicarbonate and buffer B of 60% MeCN in 10 mM ammonium bicarbonate. The separation of GSL glycans was conducted over a linear nanoLC gradient of solvent B from 1 to 65% in 80 min at a flow rate of 0.4 μL/min. After analysis, the column was washed with 85.5% MeCN in 10 mM ammonium bicarbonate for 10 min. MS spectra were acquired within a mass to charge ratio (*m*/*z*) of 340–2000 in negative ion mode with a target mass of the smart parameter setting at *m*/*z* 1200. The glass capillary voltage, dry gas temperature, dry gas flow, and nebulizer gas were set at 1000 V, 280 °C at 5 L/min, and 3 psi, respectively.

### Data Processing

The GSL glycan structures were assigned on the basis of the known MS/MS fragmentation patterns in negative ion mode^2,18,20−22^ and general glycobiology knowledge about the biosynthetic pathways of GSLs from a KEGG database.^23^ In addition, exoglycosidase digestion was used to further confirm specific linkages. Extracted ion chromatograms (EICs) were generated by extracting the theoretical mass of GSL glycans of the observed singly and doubly charged species using the first three isotopes. Peaks were manually evaluated and automatically integrated using the Data analysis software (version 5.0). The peak area-under-the-curve was obtained to integrate each peak of individual glycans if the signal-to-noise ratio was ≥6. Relative quantification was calculated on the total area of all GSL glycans within one sample normalizing to 100%.

Glycans were assigned into different glycosylation features (Table 1 and Supplementary Information, Table S-2). For further data analysis and visualization, the packages “ggplot2”, “tidyverse”, “mixOmics”,^24^ “devtools”, “MASS”, “lattice”, “BiocManager”, “tidyHeatmap”, and “complexUpset” were used in “R” software (version 4.0.5). In addition, rcc and cim functions of the “mixOmics” package were used for canonical correlation analysis and clustered image mapping in which the colored block indicates the correlation between glycosylation features and relevant gene expression data extracted from the Cancer Cell Line Encyclopedia (CCLE) database, obtained by nonstrand specific RNA sequencing using the large-scale, automated variant of the Illumina TruSeq RNA sample preparation protocol.^25^

**Table 1:**
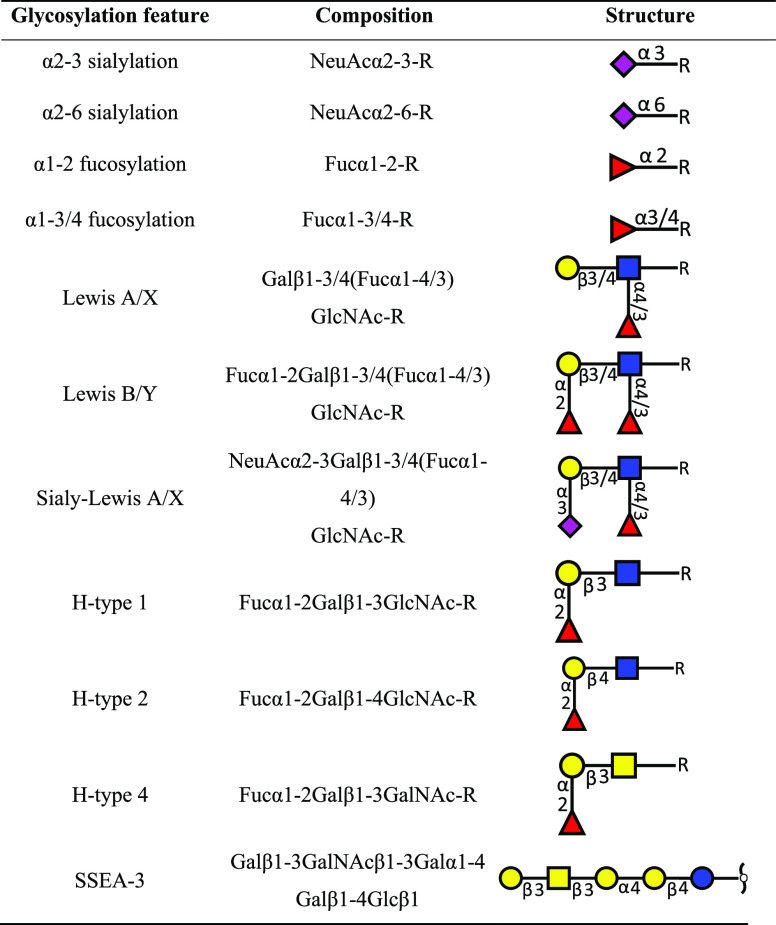
Glycosylation Features and Their Corresponding Composition and Structure with the Supplementary Information, Table S-2 Showing to which Feature Each GSL Glycan Belongs

## Results

### High Diversity of GSL Glycomic Profiles in AML Cell Lines

To characterize structural features of GSL glycans in 19 AML cell lines, GSLs were extracted from the cell lines followed by glycan head group release and analysis by PGC-nanoLC-ESI-MS/MS.^26^ A graphical representation of the whole workflow is given in the Supplementary Information, Figure S-1. In total, 79 GSL glycans were detected and characterized, and their relative abundance was calculated per cell line (Supplementary Information, Tables S-2 and S-3.1–S-3.20). To reduce the complexity of each GSL glycomic profile per cell line and define technical variability of the workflow used in our study, the single mass spectrometry average composition (MSAC) was calculated (Supplementary Information, Tables S-1 and S-4) from relative abundances.^27^ The low technical variability of the workflow is illustrated by the clustering of the two technical replicates of each cell line in a principal component analysis (PCA) (Supplementary Information, Figure S-2).

**Figure 1:**
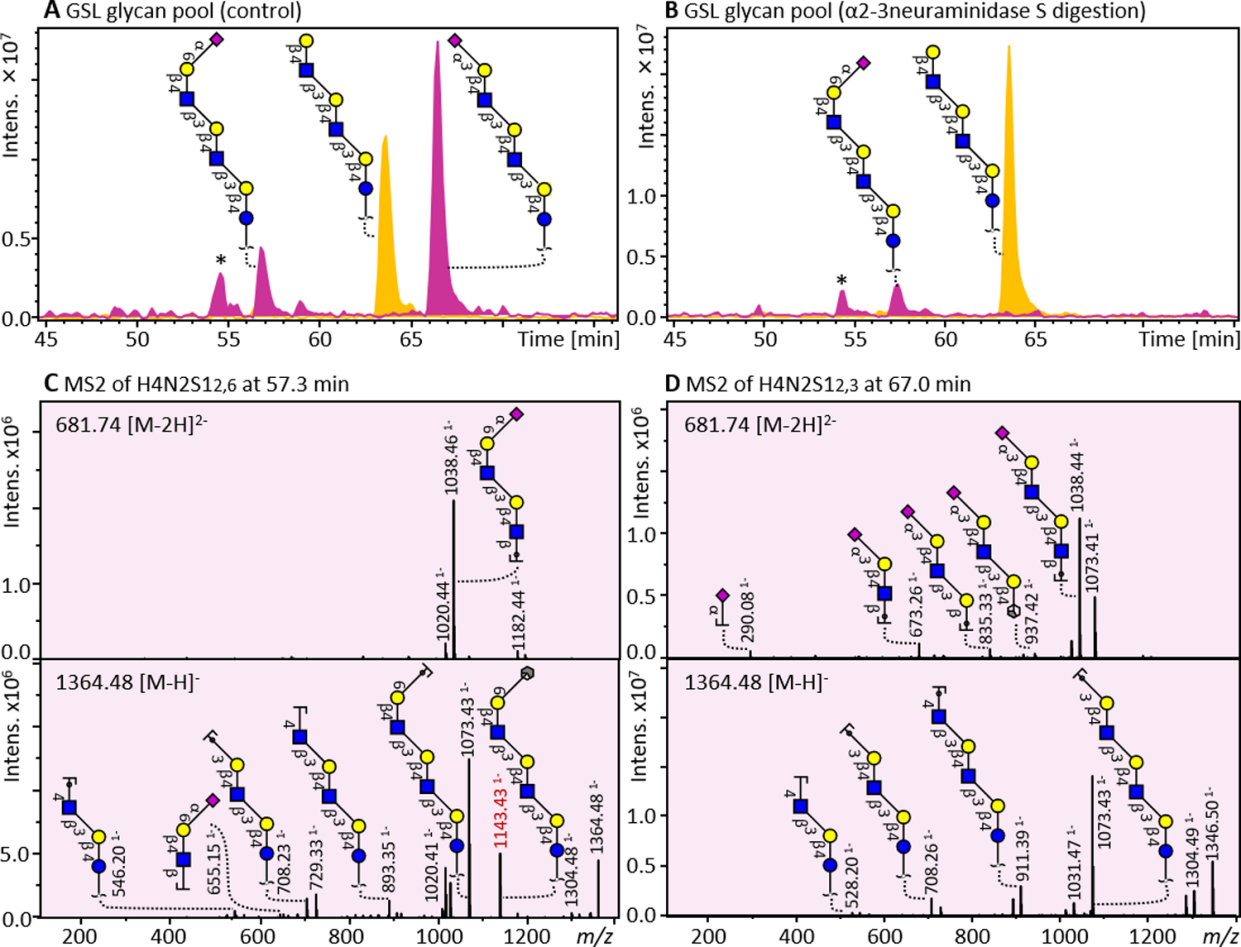
PGC-nanoLC-MS/MS analysis of GSL glycans of an AML cell line without (A) and with α2–3 neuraminidase S treatment (B). EICs represent the non-sialylated GSL glycan (H4N2; *m*/*z* 1073.39; yellow trace) and sialylated GSL glycans (H4N2S1; *m*/*z* 1364.48; pink trace). Fragmentation spectra of the two isomeric species at 57.3 and 67.0 min are illustrated in panel (C) (H4N2S1_2,6_) and (D) (H4N2S1_2,3_), respectively. MS/MS spectra of the doubly (*m*/*z* 681.742^–^) and singly charged (*m*/*z* 1364.48^–^) precursor ion are shown. “*” indicates an analyte with *m*/*z* 1365.55^–^.

The structural elucidation of GSL glycans requires a sensitive, high-resolution platform because of the heterogeneity of GSL glycans. PGC-nanoLC was used in this study because of its ability to separate isomeric glycans.^21^ Known diagnostic ions such as ^0,2^A-type and ^2,4^A-type were used to characterize C-4 substitution of the GlcNAc and Glc residues, respectively.^22^ Blood group and Lewis antigens were defined among the GSL glycans based on the presence of specific D and A diagnostic fragment ions (see Table 1 for all the specific GSL glycosylation features).^18^ Exoglycosidase treatment was conducted to further obtain partial linkage information of specific GSL glycans. For instance, two isomeric GSL glycans with the composition of H4N2S1 from a pooled sample of GSL glycans were separated by PGC (Figure 1A; highlighted in pink**)**. Upon α2–3 neuraminidase treatment, the peak at 67.0 min disappeared while the peak at 57.3 min remained thereby revealing the sialic acid linkages of these isomeric species (Figure 1B**)**. In addition, the MS/MS fragmentation spectra of the isomeric glycans provided evidence for the sialic acid linkage because of the presence of diagnostic cross-ring fragment ^0,2^X_6_ at *m*/*z* 1143.43^–^ (Figure 1C). The β1,4-substitution of GlcNAc was confirmed by the diagnostic fragments ^0,2^A_5_ at *m*/*z* 937.37 and is in agreement with previous studies.^2,21,22^

**Figure 2:**
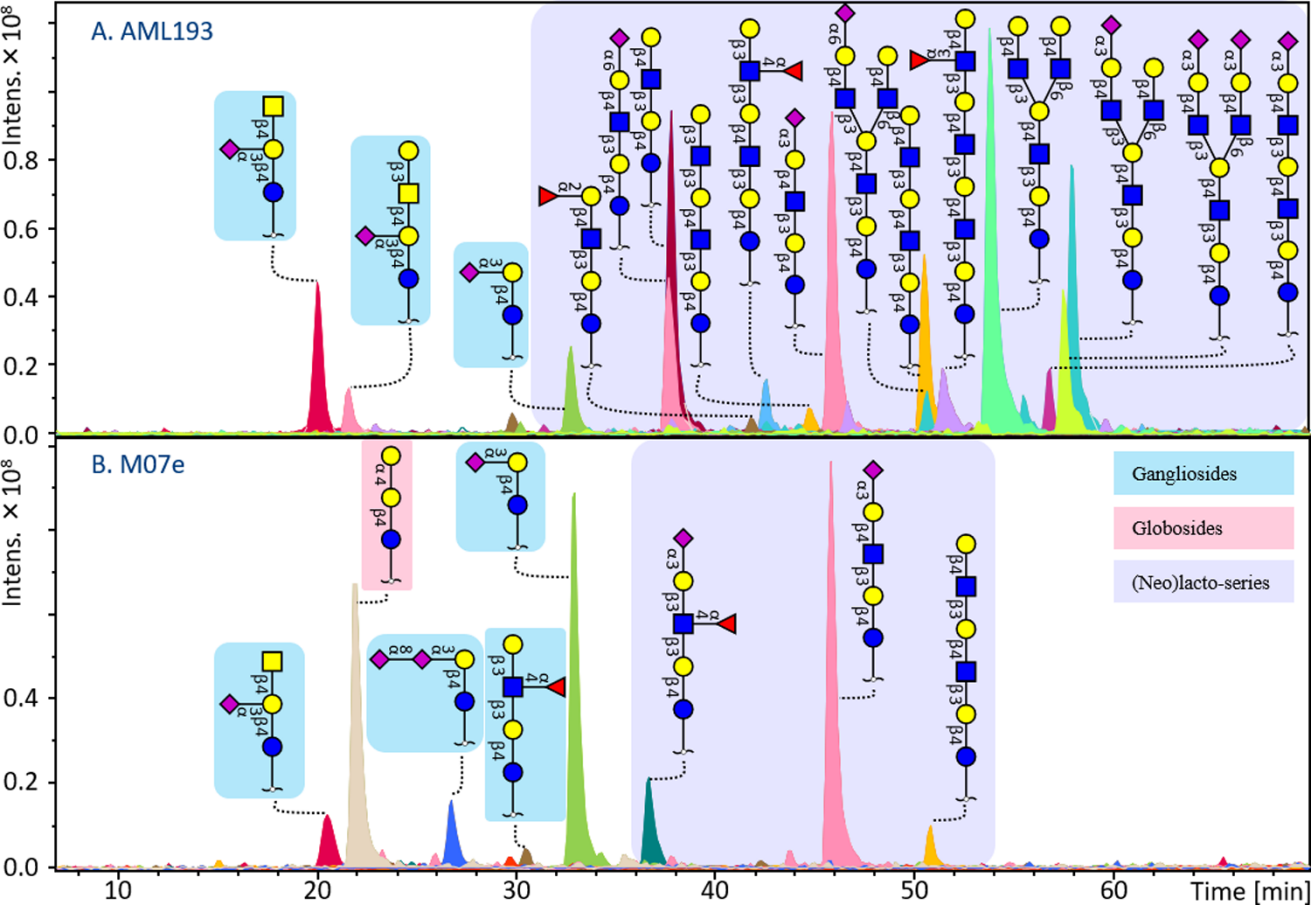
GSL glycan profiles of two exemplary AML cell lines. (A) GSL glycan profile of cell line AML 193 expresses gangliosides, and high diversity of (neo)lacto-series including linear and branched GSL glycans, with a high expression of I antigen, no globosides detected. (B) Cell line M07e reveals high abundance of gangliosides, some globosides, and (neo)lacto-series, but less diversity and no I branching expressed. The background in blue, yellow, and pink represents the gangliosides, globosides, and (neo)lacto-series glycans, respectively. Symbols of monosaccharide residues from the Symbol Nomenclature for Glycans system were used.

A high variation was observed in the GSL glycomic profiles between the 19 AML cell lines as exemplified in Figure 2 and the Supplementary Information, Figure S-3. Interestingly, the ganglioside glycan GM3 was the only common glycan present in all cell lines (Supplementary Information, Figure S-3 and Table S-5). The subtypes M2 and M4 revealed the highest diversity, showing 9 and 18 unique GSL glycans, respectively, and most of the unique glycans expressed in M4 belong to (neo)lacto-series (Supplementary Information, Figure S-4). These glycans carried blood group H or Lewis B/Y antigens (Supplementary Information, Figure S-4). However, Lewis A/X antigens were more broadly expressed in the M2/4/5 and 7 subtypes. Furthermore, three types of gangliosides and two kinds of (neo)lacto-series glycans were found across all subtypes, for more details see the Supplementary Information, Figure S-4 and Table S-5. Globoside glycans were present at low abundance in most AML cell lines but were relatively abundant in the cell lines THP1 (32%), TF1 (17%) and M07e (20%) (Supplementary Information, Figure S-5 and Table S-2). As shown in Figure 2, a high expression of (neo)lacto-series, including linear and branched glycans (I antigens), was found for cell line AML 193 (M5 subtype), whereas these glycans were rarely detected in the M07e cell line (M7 subtype) (Supplementary Information, Figure S-5). In contrast, higher expression of gangliosides as well as globosides including SSEA-3 was found in the glycan profile of cell line M07e. Interestingly, *N*-glycolylneuraminic acid (Neu5Gc) was detected in most of the AML cell lines (Supplementary Information, Table S-1).

### Association of GSL Glycosylation Traits with Cell Line Classifications

PCA was performed to discover possible associations between GSL glycosylation traits and the AML classification of the cell lines. For this, GSL glycans were grouped based upon the composition and specific structural features (Table 1 and the Supplementary Information, Table S-2). Variability in the loadings plot was assessed to elucidate which glycosylation features drive the separation in the PCA model.

**Figure 3:**
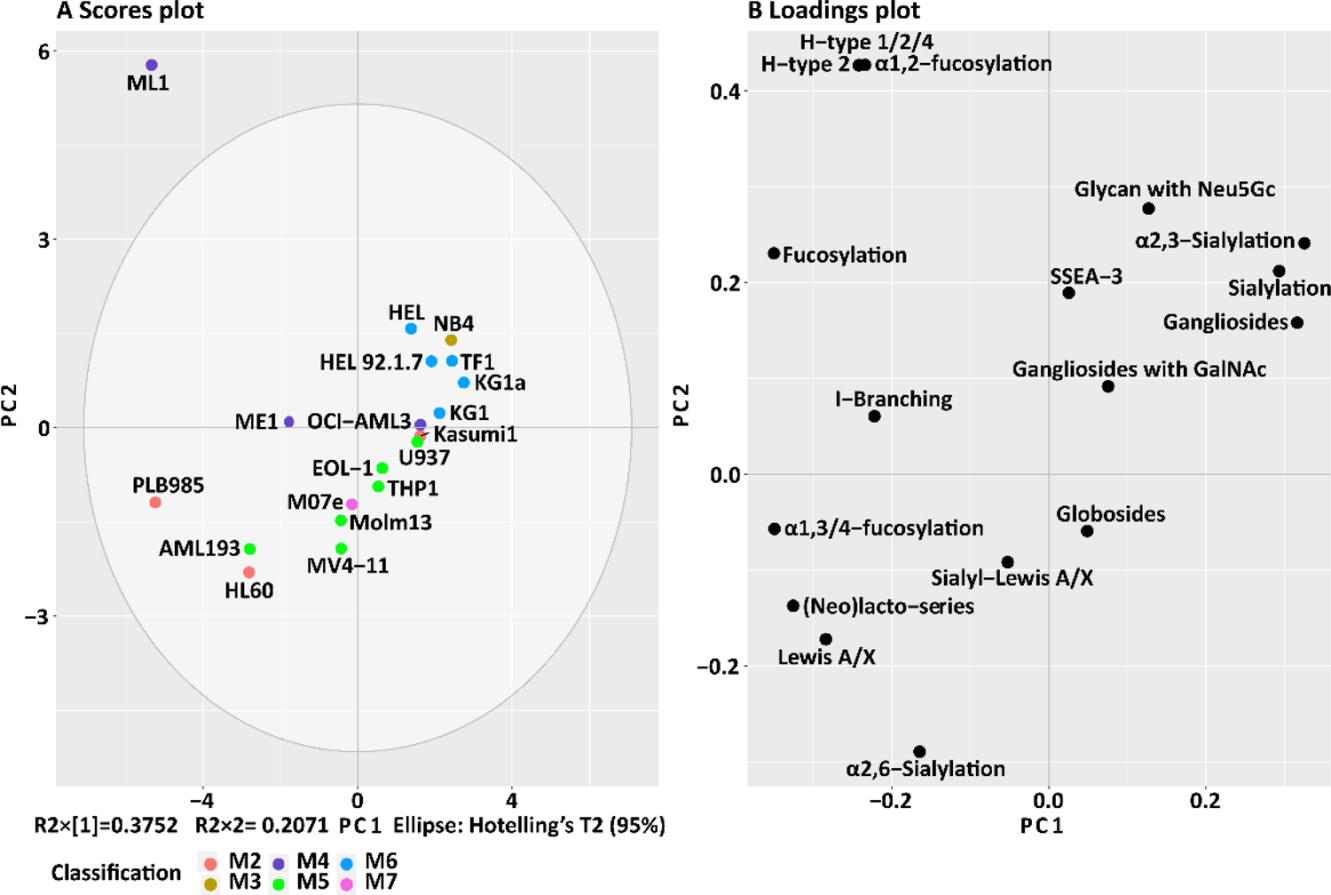
PCA of GSL glycan structural features and their relative abundance (%) in AML cell lines. (A) PCA scores plot of PC1 against PC2. (B) PCA loadings plot indicates the contribution of each glycosylation trait to the PCA model. The top two PCs explain 57.33% of the variation within the data. Technical replicates were averaged for each cell line. AML cell lines are color-coded based upon the FAB classification.

As shown in Figure 3, cell lines belonging to subtype M6 (HEL, HEL92.1.7, KG1, KG1a, and TF1) cluster together in the upper right part of the scores plot because of the high expression of gangliosides (average 70.2% for these five cell lines *vs* the average abundance of 37.2% for the other 14 AML cell lines analyzed) and sialylation, including α2,3-sialylation and glycans occupied with Neu5Gc (Supplementary Information, Figure S-4 and Table S-6). The expression levels of gangliosides, sialylation, α2,3-sialylation, and Lewis A/X antigen in AML cell lines can be found in the Supplementary Information, Figure S-6. A similar glycosylation profile with additional high expression of gangliosides with GalNAc was observed for the M3 subtype cell line NB4, which is the only M3-classified cell line in our panel. Cell lines belonging to the M5 subtype (AML193, MV4–11, Molm13, THP1, EOL-1, and U937) are mainly grouped in the bottom region of the PCA scores plot because of the higher expression of (neo)lacto-series, sialyl-Lewis A/X, and Lewis A/X (Figure 3). High expression of α2,6-sialylation and (neo)lacto-series plays an essential role in the clustering of cell lines AML193, MV4–11, and Molm13. These three M5 cell lines AML193, MV4–11, and Molm13 showed an average level of *α*2,6-sialylation of 17.4 *vs* 3.9% as an average for all the other AML cell lines analyzed. An average relative abundance of 76.9% was found for the (neo)lacto-series expression of M5 cell lines *vs* 44.7% for the other 16 AML cell lines (Supplementary Information, Table S-6). In contrast, cell line U937 – albeit also of the M5 class – showed very low expression of (neo)lacto-series with high expression of gangliosides, and cell line THP1 indicated a specifically high expression of globosides. No consistency was found for the M4 subtypes with regard to GSL glycan feature expression. However, it should be noted that a uniquely high expression of blood group H-type antigens was found in the ML1 cell line. Two cell lines belonging to subtype M2 (PLB985 and HL60) also show high (neo)lacto-series expression (80.0 and 92.9%, respectively), notably accompanied by high expression of Lewis A/X type antigen (19.1 and 5.1%, respectively) (Supplementary Information, Table S-6). HL60 has a high expression of α2,6-sialylation while the third M2 cell line, Kasumi1, appears to have a distinct profile from the other two, revealing a high abundance of gangliosides, sialylation, and α2,3-sialylation. The M07e cell line, the only M7-classified cell line in our panel, shows specifically high expression of sialyl-Lewis A/X antigens together with higher expression of globosides as shown in Figure 2B.

### Association of Glycosylation Features with the Transcriptional Profiles in AML Cell Lines

**Figure 4:**
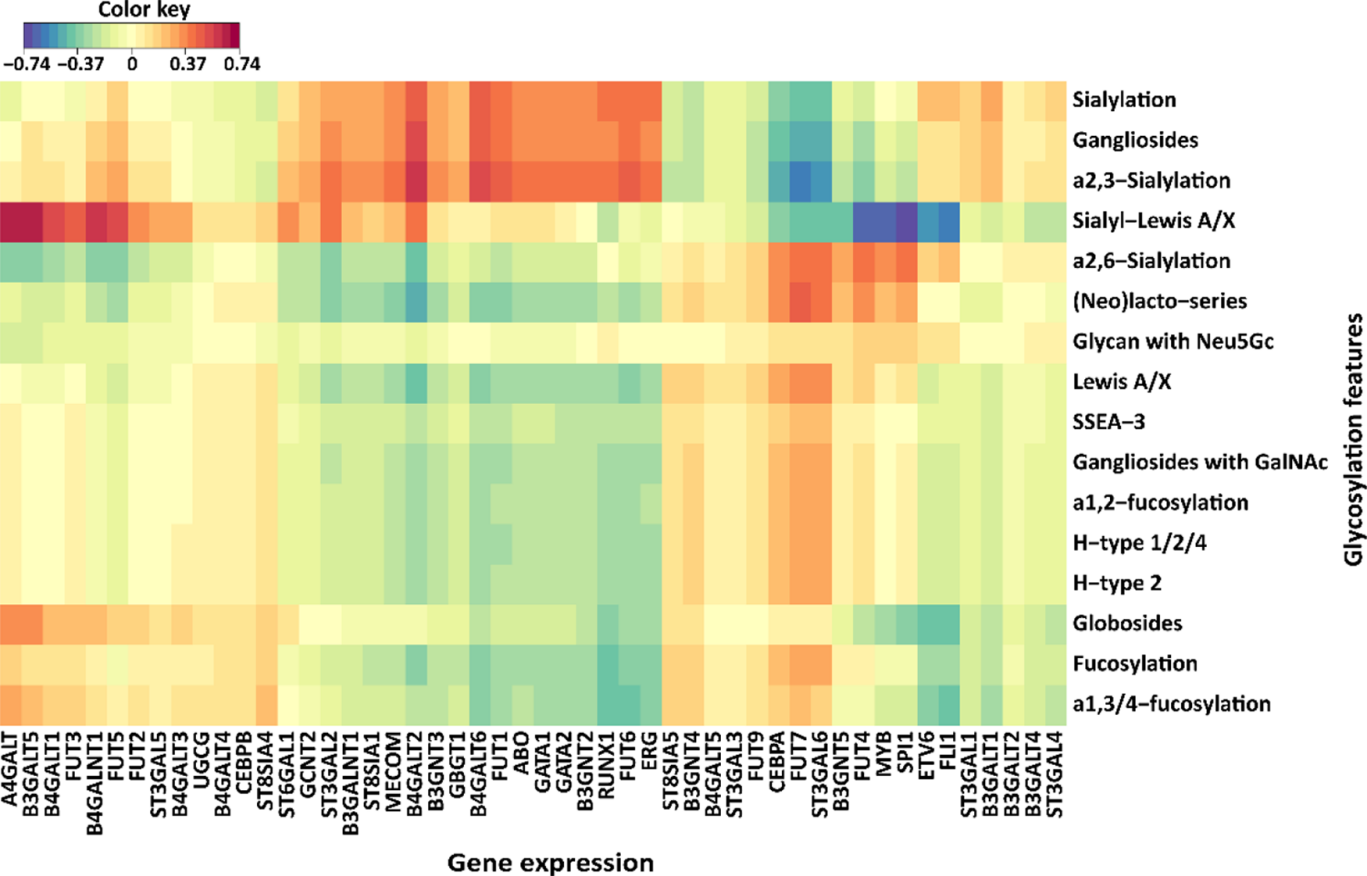
Associations of GSL glycan structural features with gene expression of GTs and hematopoietic TFs. The clustered heat map of the canonical model illustrates the correlation between glycosylation features and gene expression of corresponding GTs and TFs. The canonical correlation analysis was conducted based on the dataset of relative quantification of the glycosylation features (right) in 17 AML cell lines and the dataset of gene expression of relevant GTs and TFs (bottom) which were extracted from the CCLE. The correlation is indicated at the top legend (blue: negative correlation; red: positive correlation).

**Figure 5:**
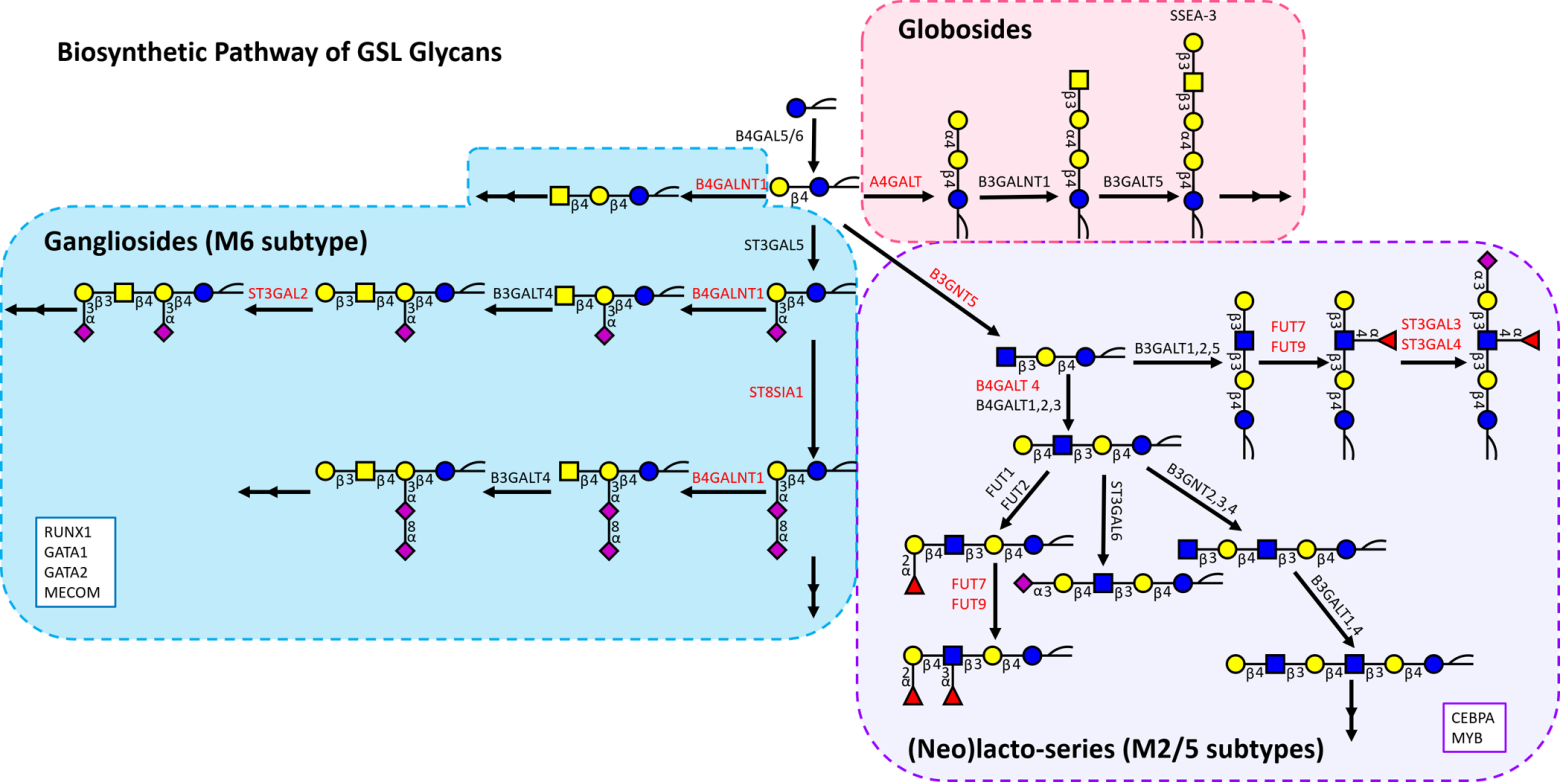
Biosynthetic pathway of GSLs with related genes encoding for the GTs involved in the biosynthesis. The three main GSL groups of globosides, (neo)lacto-series, and gangliosides are highlighted in pink, purple, and blue, respectively. GTs that showed a positive correlation with a relevant GSL group or glycosylation trait are given in red. TFs in the boxes showed a positive correlation with the corresponding group of GSL glycans. Double arrows imply the further elongation of GSLs.

Cellular glycosylation signatures are known to be largely determined by GSTs on the one hand, and various TFs on the other hand, as indicated by various studies using cell lines as model systems, with glycosylation changes often reflecting cellular differentiation.^28−31^ To explore the associations between the expression of GSL glycans and the expression of a preselected set of genes including GTs involved in GSL biosynthesis and TFs involving the differentiation of the blood cells, the transcriptomic data of the same AML cell lines were extracted from the CCLE.^25^ The selection of hematopoietic TFs was based on their key roles in the regulation of the process of blood cell differentiation and maturation and their dysregulation involved in the development of AML.^32−35^ Canonical correlation analysis was performed between expression of GSL glycan traits as listed in Table 1 and selected genes (Figures 4 and 5). The positive correlations of GSL glycans with GTs were found as follows: The relative abundance of (neo)lacto-series structures was found to positively correlate with *B3GNT5* expression, a gene that codes for the only enzyme that drives (neo)lacto-series biosynthesis (*r* = 0.38). Likewise, the relative abundance of globosides correlated with *A4GALT* gene expression coding for the first enzyme specific for the globoside biosynthetic pathway (*r* = 0.38). A subtle correlation was found between the gene that is involved in the ganglioside series, *B4GALNT1* (*r* = 0.21) and the expression of gangliosides. The expression of the fucosyltransferases *FUT7* and *FUT9* encoding genes correlated positively with the fucosylated Lewis A/X antigen (*r* = 0.30 and 0.27, respectively) and α1,3/4-fucosylation (*r* = 0.13 and 0.22, respectively). Similarly, a positive correlation was found between the *α*2,3-sialyltransferase encoding gene *ST3GAL2* and *α*2,3-sialylation (*r* = 0.24) and between *ST8SIA1* and sialylation (*r* = 0.30) and gangliosides (*r* = 0.32).

To elucidate the potential regulation of GSL glycan expression, the correlations between GSL glycosylation features as listed in Table 1 and the expression of hematopoietic TFs were explored. As shown in Figure 4, TFs *GATA1*, *GATA2*, *RUNX1,* and *MECOM* showed positive correlations with gangliosides (*r* = 0.36, 0.31, 0.28, and 0.37, respectively) and sialylation (*r* = 0.37, 0.36, 0.39, and 0.34, respectively). Meanwhile, *GATA1* presented strong positive correlations with *ST3GAL2* and *ST8SIA1* (*r* = 0.69 and 0.79, respectively), In addition, high positive correlations were found between *MECOM* with *ST3GAL2* (*r* = 0.75) and *ST8SIA1* (*r* = 0.85) as well as between *RUNX1* with *ST3GAL2* (*r* = 0.40) and *ST8SIA1* (*r* = 0.40) (Supplementary Information, Figure S-7). These findings revealed that *GATA2*, *GATA1*, *MECOM,* and *RUNX1* might play parts in the high expression of sialylation within the M6 subtype by regulation of corresponding GTs *ST3GAL2* and *ST8SIA1*.

For the (neo)lacto-series, the TFs, *CEBPA* (*r* = 0.14) and *MYB* (*r* = 0.22), showed a moderate positive correlation. Interestingly, *B3GNT5* responsible for production of (neo)lacto-series exhibited strong positive correlations with *CEBPA* (*r* = 0.54) and *MYB* (*r* = 0.39), respectively (Supplementary Information, Figure S-7). Additionally, TFs *CEBPA* and *MYB* showed positive correlations with *FUT7* (*r* = 0.84 and 0.31, respectively) which were positively correlated with the Lewis A/X antigen structure and α1,3/4-fucosylation as shown above. Thus, our in-depth analyses show that several relations exist between the expression of specific GTs and TFs and the biosynthesis of GSL glycans.

## Discussion

GSLs are involved in many cellular processes including cellular interaction, differentiation, signal transduction, and oncogenesis.^36^ The structural and functional classification of GSLs is mainly based on the glycan part. Little is known about the expression and regulation of GSLs in AML cells because isolation and characterization of the GSLs is a challenging task due to the heterogeneity and isomeric complexity present in the glycan constituents. In this study, a PGC-nanoLC-ESI-MS/MS platform was employed to detect, identify, and quantify the glycan head group of GSLs extracted from AML cell lines. The broad specificity enzyme EGCase I was used for releasing GSL head groups, allowing to profile all human GSL series in a single analysis.^37^ In total, 79 GSL glycans were detected and characterized. An overall high diversity in GSL glycan profiles was found for the 19 AML cell lines.

Previous studies indicated that incomplete biosynthesis and neosynthesis are the two main mechanisms that underlie aberrant glycan expression in hematological malignancies because of the alteration of transcription of GTs and/or glycosidase gene(s).^7,38^ In line with these reports, we found associations between gene expression and GSL expression in 19 AML cell lines. The expression of (neo)lacto-series correlated positively with the expression of *B3GNT5* which encodes the key enzyme involved in the biosynthesis of (neo)lacto-series GSLs(Figures 4 and 5). Of note, (neo)lacto-series GSL synthesis is initiated by *B3GNT5* which recently has been found to be strongly regulated by the protease SPPL3 at the post-translational level pointing toward an important role of (neo)lacto-series GSLs in the inhibition of immune recognition.^39^ Accordingly, one may speculate about the potential immunosuppressive roles of (neo)lacto-series GSLs in AML. Meanwhile, both globosides and SSEA-3, which is a surface marker of human embryonic stem cells^40^ and human-induced pluripotent stem cells,^41^ showed positive correlations with *A4GALT* which encodes the corresponding GT responsible for the biosynthesis of globoside glycans. Similarly, the expression of *B4GALNT1* encoding for a key enzyme in ganglioside biosynthesis positively associated with ganglioside levels.

Fucosylation, which is a nonextendable modification, plays a critical role in the homing of hematopoietic cells to the bone marrow.^7^*FUT7* and *FUT9* are known for their involvement in the biosynthesis of α1,3- and α1,4-linked fucosylation, which is in agreement with our findings that *FUT7* and *9* show positive correlations with the levels of α1,3/4-fucosylation (Figure 4). Of note, while the role of the *FUT7* enzyme in the synthesis of sialyl-Lewis X has been demonstrated in a previous study,^42^ a strong negative correlation between *FUT7* and sialyl-Lewis A/X was observed in our data. In another report, *FUT7* has been found to be involved in the synthesis of the Lewis antigen,^43^ and we accordingly found a positive correlation of *FUT7* expression with the levels of Lewis A/X antigens which were particularly high in cell lines AML193, PLB985, and HL60, all belonging to the myelocytic lineage (Supplementary Information, Figure S-4 and Table S-1). Our data imply that the expression of FUT7 may play a role in the synthesis of Lewis A/X antigens rather than sialyl-Lewis A/X. In addition, the finding of a previous study of increased Lewis A/X antigens on *N*- and *O*-glycome in the M2 and M5 subtypes is consistent with our current result.^5^ In contrast, blood group H antigens were detected in cell lines originating from myelocytes/erythrocytes/monocytes. These H antigens are highly expressed in ML1 and ME1 (M4 subtype belonging to myeloblasts). Additionally, Lewis B/Y antigens which are potential therapeutic targets due to the high expression in various cancers were only detected in ML1 and ME1 cell lines (Supplementary Information, Figure S-4 and Table S-1).^44^ Therefore, Lewis B/Y antigen might be a potential target for M4 subtype AML. To further corroborate this, AML cells obtained from AML patients with the M4 subtype could be interesting study objects for glycomics analysis.

Because of a deletion of the gene *cmah*, Neu5Gc is absent in human glycans.^45^ However, GSL glycans with Neu5Gc were found in most AML cell lines in this study, including GM3 (Neu5Gc), GD3 (Neu5Gc), GM1a (Neu5Gc), and two glycans in the (neo)lacto-series. The abundance of glycans with Neu5Gc varied between cell lines with an average Neu5Gc expression of 2.3% in M6 cell lines *vs* only 0.8% in the rest of the AML cell lines (Supplementary Information, Table S-6). Several studies have suggested that increased levels of Neu5Gc may be attributed to the higher metabolic rate in malignant cells,^46^ the induction of sialic acid transporters under hypoxic conditions, and dietary incorporation.^47−49^ We suspect that the Neu5Gc found in the studied AML cell lines may have been incorporated from the culturing medium or its biosynthesis might originate from the mutation of relevant genes in AML cell lines. Previous studies revealed the expression of Neu5Gc in humans being associated with malignant transformation, becoming, therefore, an attractive target for cancer immunotherapy.^50^ Therefore, the glycans with Neu5Gc may be potential therapeutic targets for M6-stage AML. For a better understanding of the underlying mechanism of expression of glycans with Neu5Gc in AML cell lines, more studies are needed.

A recent study revealed that I blood group antigens (I-branched glycans), which are initiated by the addition of a GlcNAc to internal residues of poly-LacNAc chains in β1,6 linkage, played a critical role in cancer progression through the regulation of malignancy-associated adhesive and metastatic activities.^51^ I antigens were first identified on human RBCs and have a reciprocal relationship with i antigens.^52^ Adult human RBCs fully express I antigens and contain only few i antigens, and alteration of expression patterns of I and i antigens has been found during oncogenesis.^52^ In this study, the expression of I antigens was also found in most of the cell lines belonging to the subtypes M2, M4, and M5. Interestingly, all the cell lines expressing I antigens – except for U937 – were initially derived from peripheral blood (Supplementary Information, Table S-1). In addition, the cell lines HEL92.1.7, KG1, KG1a, TF1, and NB4 clustered well in the scores plot in PCA analysis and were all obtained from bone marrow (Supplementary Information, Table S-1), which indicates that the expression of certain GSLs may reflect the origin of the cell lines.

A previous study has indicated that mutation, translocation, and aberrant expression of specific TFs can result in malignant transformation of hematopoietic cells.^34^ Accordingly, certain hematopoietic TFs such as *CEBPA*,^53^*MYB,*^54^ and *RUNX1*(55) have been considered as therapeutic targets and hematopoietic TFs are known to be involved in the differentiation and oncogenesis of blood cells.^32^ TF *GATA2* is essential for hematopoietic differentiation and lymphatic formation^35^ and considered as a marker for AML with poor prognosis because of a higher expression in AML than in normal bone marrow.^56^ In this study, we found that *GATA2* was highly expressed in cell lines of the M6 subtype (Supplementary Information, Figure S-8) and the expression of *GATA2* correlated positively with sialylation, α2,3-sialylation, and gangliosides (Figure 4) which were highly expressed in the M6 subtype (Figure 3). Our analyses further revealed that *GATA2* correlated with *ST3GAL2* (*r* = 0.66) responsible for addition of sialic acid to terminal galactose of gangliosides and with *ST8SIA1* (*r* = 0.66) gene expression encoding for an *α*2,8-sialyltransferase which acts on sialic acid residue by addition of another sialic acid (Supplementary Information, Figure S-7). Therefore, *GATA2* might be involved in the upregulation of ganglioside expression and sialylation in the M6 subtype. We speculate that poor prognosis of AML might be linked to the high expression of α2,3-sialylation, sialylation, and gangliosides regulated by *GATA2* which is highly expressed in M6-subtype cell lines. Our data point toward a shared effect of certain TFs on different types of glycoconjugates, as *GATA2* likewise shows positive correlation with the total levels of sialylation on *N*- and *O*-glycome in AML cell lines.^5^ Meanwhile, erythrocyte/megakaryocyte-related TF *GATA1*(57) has been found to be expressed at low levels in M3, M4, and M5 AML.

High expression of *MECOM* and *GATA1* has been found in several erythrocyte-derived M6 subtype cell lines (HEL, HEL92.1.7, and TF1) and the megakaryocyte-derived M07e cell line (Supplementary Information, Figure S-8). Interestingly, *GATA1*, *MECOM,* and *RUNX1* expression positively correlated with sialylation (*α*2,3-sialylation and gangliosides). Strong positive correlations were observed for *GATA1* with *ST3GAL2* and *ST8SIA1* (*r* = 0.69 and 0.79, respectively), for *MECOM* with *ST3GAL2* and *ST8SIA1* (*r* = 0.75 and 0.85, respectively) as well as for *RUNX1* with *ST3GAL2* and *ST8SIA1* (*r* = 0.40 and 0.40) (Supplementary Information, Figure S-7). These findings indicate that *GATA2*, *GATA1*, *MECOM,* and *RUNX1* may be involved in the high expression of sialylation in the M6 subtype by regulation of the corresponding GTs *ST3GAL2* and *ST8SIA1*. Taking into account the previous findings, TF *GATA2* may play an essential role in the regulation of sialylation of various glycoconjugates in M6 cell lines. To confirm this, knockout, knockdown, and overexpression experiments targeting *GATA2* in M6 cell lines assessing the effect on various glycoconjugates would be informative.^5^

*CEBPA* participates in the differentiation of common myeloid progenitors into basophils,^32^ and its mutation is associated with AML.^58^ Additionally, *MYB* plays a key role in the hematopoietic system and has been recognized as an attractive therapeutic target for the treatment of leukemia.^59^ Both TFs (*CEBPA* and *MYB*) are mainly expressed in subtypes M2 and M5 of the AML cell lines which are characterized by high expression of (neo)lacto-series and Lewis A/X antigens (Supplementary Information, Figure S-8). Our study revealed a modest positive correlation for TFs *MYB* and *CEBPA* with (neo)lacto-series structures (*r* = 0.22 and 0.14, respectively) (Figure 4) and the corresponding key GT gene *B3GNT5* (*r* = 0.39 and 0.54) (Supplementary Information, Figure S-7). As mentioned above, the Lewis A/X antigen structure and α1,3/4-fucosylation were positively associated with *FUT7* (Figure 4) which also showed a positive correlation with TFs *CEBPA* (*r* = 0.84) and *MYB* (*r* = 0.31) (Supplementary Information, Figure S-7). The results indicate that the hematopoietic TFs *CEBPA* and *MYB* may play critical roles in expression of GSL glycan structures ((neo)lacto-series, Lewis A/X and α1,3/4-fucosylation) within AML classifications M2 and M5 by regulation of the expression of GTs *B3GNT5* and *FUT7* (Figure 5). Interestingly, a positive correlation was also found between *CEBPA* and Lewis A/X of *N*- and *O*-glycome in M2 and M5 AML cell lines,^5^ which further supported the assumption of the role of *CEBPA* in the expression of Lewis A/X of glycans in M2 and M5 subtypes.

It is worth mentioning that the cell lines KG1 and KG1a were classified as different FAB subtypes in several studies.^60−62^ In this study, KG1 and KG1a were both assigned as the M6 subtype which is in agreement with BioSample in NCBI.^63^ Prominent differences in GSL glycosylation features have been observed in AML cell lines. A previous study has suggested that experimental conditions such as different culture media and labs may contribute to the different profiles of GSL glycans.^29^ In contrast, a previous study on different cell lines has shown only minor differences of N-glycosylation profiles obtained upon with two different media and in two different laboratories.^30^ Based on the findings, we assume that glycosylation may be modestly affected by the choice of the medium. To validate this, more experiments needed to be performed, particularly focusing on GSL glycosylation.

In summary, the expression of genes *B4GALNT1*, *A4GALT,* and *B3GNT5* (encoding key GTs for the biosynthesis of the three main GSL subgroups (gangliosides, globosides, and (neo)lacto-series, respectively) positively correlated with the expression of gangliosides, globosides, and (neo)lacto-series, respectively (Figure 5). In addition, the expression of *ST3GAL2*/*3*/*4* positively correlated with α2,3-sialylation and sialylation. Lewis A/X and α1,3/4-fucosylation showed positive correlations with FUT7 and FUT9, respectively. Moreover, certain hematopoietic TFs may play essential roles in the association of GSL glycans with AML classifications by regulating the related GTs as shown in Figure 5.

Our current work is fully descriptive in nature, providing a detailed map of GSL glycan structures in AML cell lines. The multiple, reasonably strong correlations between glycan species and GT and TF transcriptomic data lead to new hypotheses regarding the regulation of the GSL repertoire which will benefit from experimental confirmation. This will be the subject of future studies, with implications for cancer biology. Likewise, the analysis of patient materials using a similar approach is warranted, the main challenge being to obtain patient AML cells of sufficient purity for deriving disease glycomics signatures.

## Conclusions

In this study, a striking diversity in expression of GSL glycans was found between different AML cell lines. The biosynthesis of GSL glycans is regulated by specific GTs, some of which were confirmed at a transcriptional level. Additionally, we found new associations of GSL glycans with AML classifications. These are potentially generated by GTs whose expression is under the control of a subset of TFs. Our study shows that the hematopoietic TFs *GATA2*, *GATA1*, *MECOM,* and *RUNX1* potentially play a role in the high expression of gangliosides, sialylation, and α2,3-sialylation within the M6 subtype via regulation of GTs *ST3GAL2* and *ST8SIA1*. The hematopoietic TFs *CEBPA* and *MYB* may play decisive roles in determining the expression of GSL glycans within AML classifications M2 and M5 by regulating the related GTs *B3GNT5* and *FUT7*. Finally, the exploration of expression and regulation of GSL glycans in AML cell lines paves the way for future studies on the functional role of such GSL glycans. The enzymes in control of their production may provide novel therapeutic targets for specific AML subtypes.

## Supporting Information Available

Figure S1: Workflow for the analysis of GSL-derived glycans from cell lines by PGC-nanoLC-MS/MS; Figure S2: The technical variation is illustrated by the close clustering of the technical replicates for each cell line and system suitability standard mixture; Figure S3: Structural diversity of GSL glycans in AML cell lines; Figure S4: The UpSet plots display the intersections between AML cell lines grouped by classes and (A) GSL glycan subgroups, (B) glycosylation traits, and (C) glycan antigens, respectively; Figure S5: Distribution of GSL glycans features (derived traits) in specific AML cell lines illustrated in the heatmap; Figure S6: The distribution of glycosylation features in AML cell lines grouped by FAB classification; Figure S7: Canonical correlation analysis between gene expression of GTs and hematopoietic TFs; Figure S8: Distribution of gene expression of hematopoietic TFs in specific AML cell lines illustrated in the heatmap (PDF)
Table S1: Characteristics of cell lines used in this study. Table S2: Overview of individual GSL glycans present in AML cell lines, with their relative abundances per cell line and abundance per glycosylation feature. Table S3: All the observed GSL glycans per cell line. Retention times (min), peak areas, relative abundances with their corresponding standard deviation, and relative standard deviation (CV) are provided as well as the calculations regarding monosaccharide average compositions for each technical replicate (1–2 replicate). Table S4: Calculated MSACs per technical replicate (1 or 2). Table S5: Overview of individual GSL glycans present in AML cell lines, with their retention time (min), relative abundances per cell line, standard deviation, and relative standard deviation (CV). Table S6: Average expression of glycan features in centain AML cell lines. Table S7: Data for Figure 3. Table S8: Data for Figure 4. Table S9: Data for supplementary Figure 2. Table S10: Data for supplementary Figure S3. Table S11: Data for supplementary Figure S4. Table S12: Data for supplementary Figure S5. Table S13: Data for supplementary Figure S6. Table S14: Data for supplementary Figure S7. Table S15: Data for supplementary Figure S8 (XLSX)

## Notes

The raw mass spectrometric data files supporting the findings of this study are available in GlycoPOST in mzXML format, with the identifier GPST000238, accessible via the following link: https://glycopost.glycosmos.org/preview/8801144661e9ad3a218a3 with pin code 8159.

## Abbreviations

AML: acute myeloid leukemia
BSA: bovine serum albumin
CCLE: cancer cell line encyclopedia
Cer: ceramide
EGCase I: endoglycoceramidase I
FAB: French–American–British
FCS: fetal calf serum
Gal: galactose
Glc: glucose
GlcNAc: *N*-acetylglucosamine
GM-CSF: granulocyte-macrophage colony-stimulating factor
GSL: glycosphingolipid
GT: glycosyltransferase
IMDM: Iscove’s modified Dulbecco’s medium
MeCN: acetonitrile
MeOH: methanol
Neu5Gc: *N*-glycolylneuraminic acid
NeuAc: *N*-acetylneuraminic acid
PCA: principal component analysis
PGC-nanoLC–ESI-MS/MS: porous graphitized carbon nanoliquid chromatography electrospray ionization mass spectrometry
RP-SPE: reverse-phase solid-phase extraction
RT: room temperature
SPE: solid-phase extraction
TF: transcription factor
TFA: trifluoroacetic acid
