# Supplementary material for: Glycosphingolipid-Glycan Signatures of Acute Myeloid Leukemia Cell Lines Reflect Hematopoietic Differentiation
Source: J Proteome Res. 2022 Feb 16;21(4):1029–40. doi: 10.1021/acs.jproteome.1c00911 (PMC8981326; doi:10.1021/acs.jproteome.1c00911)
Supplement: Supplementary file 1 — pr1c00911_si_001.pdf [file pr1c00911_si_001.pdf]

Supplementary Material

Glycosphingolipid-glycan signatures of acute myeloid leukemia cell lines reflect hematopoietic differentiation

Di Wang [1], Tao Zhang [1], Katarina Madunić [1], Antonius A. de Waard [3,4], Constantin Blöchl [1, 5], Oleg A. Mayboroda [1], Marieke Griffioen [2], Robbert M. Spaapen [3,4], Christian G. Huber [5], Guinevere S.M.Lageveen-Kammeijer [1], Manfred Wuhrer [1,*]

[1] Center for Proteomics and Metabolomics, Leiden University Medical Center, Postbus 9600, 2300 RC Leiden, The Netherlands
[2] Department of Hematology, Leiden University Medical Center, Postbus 9600, 2300 RC Leiden, The Netherlands
[3] Department of Immunopathology, Sanquin Research, Amsterdam, 1066 CX, The Netherlands
[4] Landsteiner Laboratory, Amsterdam UMC, University of Amsterdam, Amsterdam, 1066 CX, The Netherlands
[5] Department of Biosciences, University of Salzburg, Hellbrunnerstrasse 34, 5020 Salzburg, Austria
* Correspondence: m.wuhrer@lumc.nl, *tel: +31(0)715266989*

Table of contents

Figure S1: Workflow for the analysis of glycosphingolipids (GSLs)-derived glycans from cell lines by PGC-nanoLC-MS/MS

Figure S2: The technical variation is illustrated by the close clustering of the technical replicates for each cell line and system suitability standard mixture

Figure S3: Structural diversity of GSL glycans in AML cell lines

Figure S4: The UpSet plots display the intersections between AML cell lines grouped by classes and (A) GSL glycan subgroups, (B) glycosylation traits, and (C) glycan antigens, respectively

Figure S5: Distribution of GSL glycans features (derived traits) in specific AML cell lines illustrated in the heatmap

Figure S6: The distribution of glycosylation features in AML cell lines grouped by FAB classification

Figure S7: Canonical correlation analysis between gene expression of glycosyltransferases and hematopoietic transcription factors

Figure S8: Distribution of gene expression of hematopoietic transcription factors in specific AML cell lines illustrated in the heatmap

Figure S-1

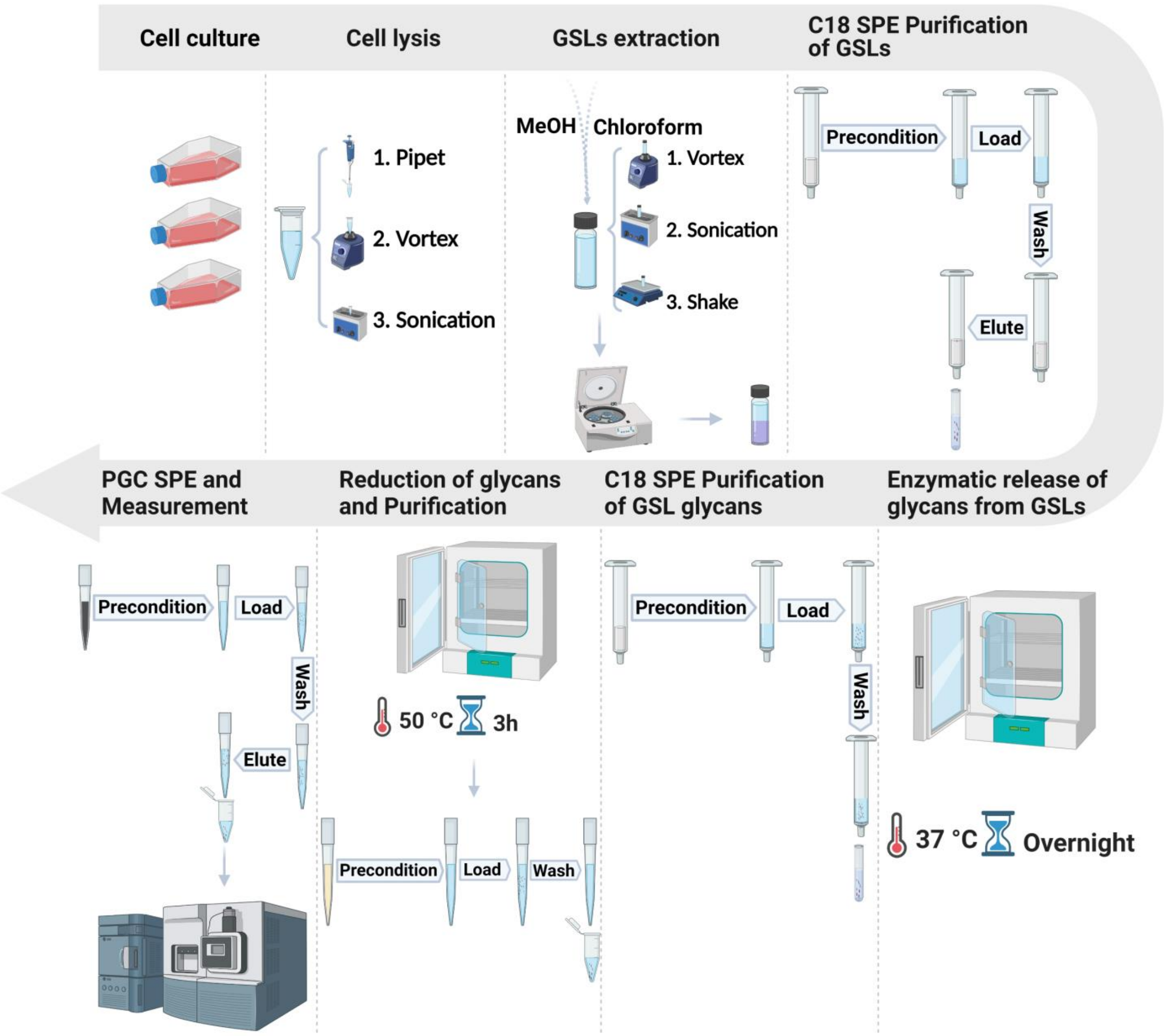

Supplementary Figure S-1. Workflow for the analysis of glycosphingolipids (GSLs)-derived glycans from cell lines by PGC-nanoLC-MS/MS. *Created with BioRender.com*.

Figure S-2

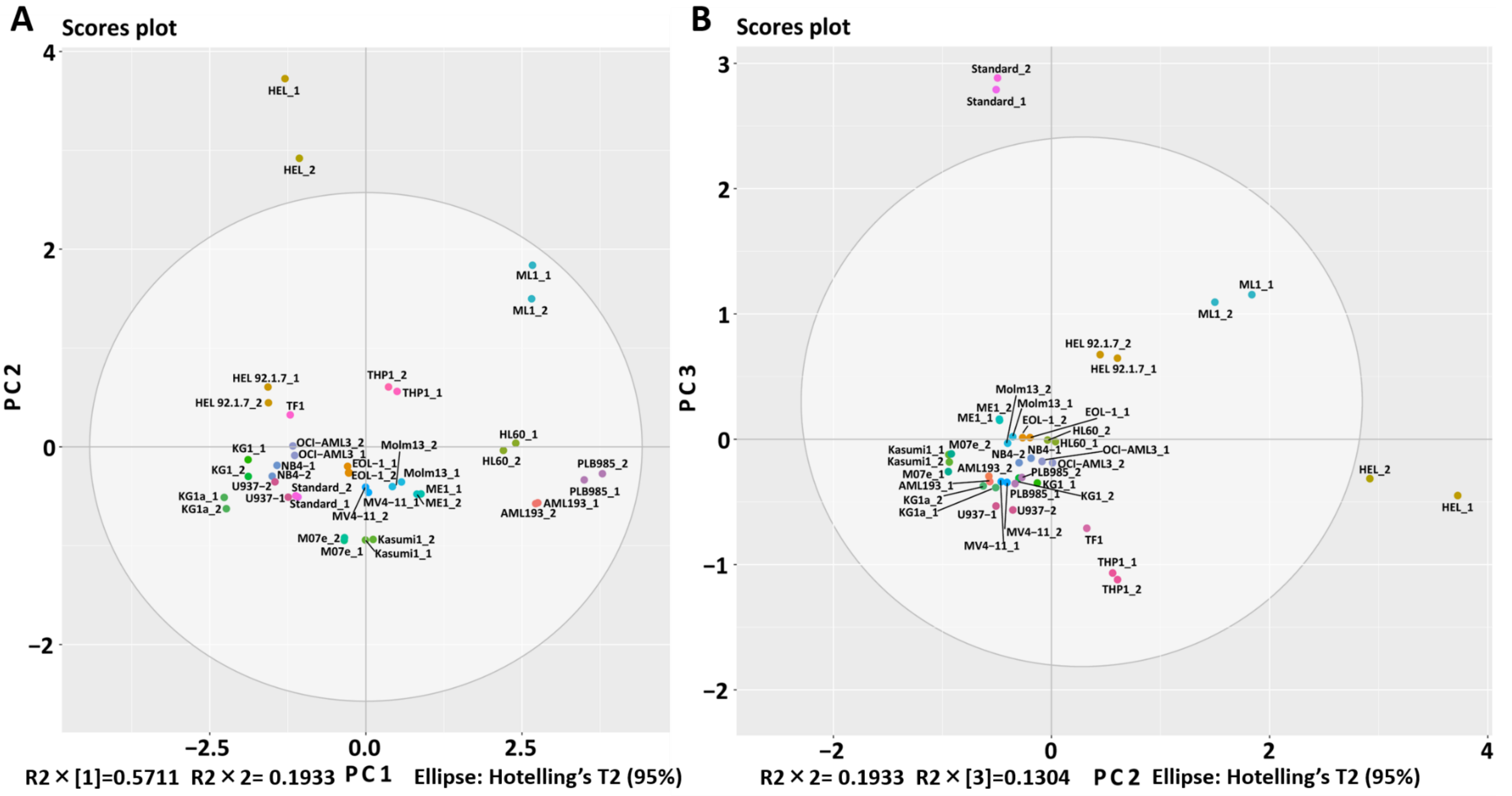

Supplementary Figure S-2. The technical variation is illustrated by the close clustering of the technical replicates for each cell line and system suitability standard mixture. (A) PC1 and PC2 scores plot and**(B)** PC2 and PC3 scores plot are based on the monosaccharide average compositions of all technical replicates (see **Supplementary Table S-1**). The robustness of the method is illustrated by the close clustering of scores. The top three principle components explain 89.48% of the variation within the data.

Figure S-3

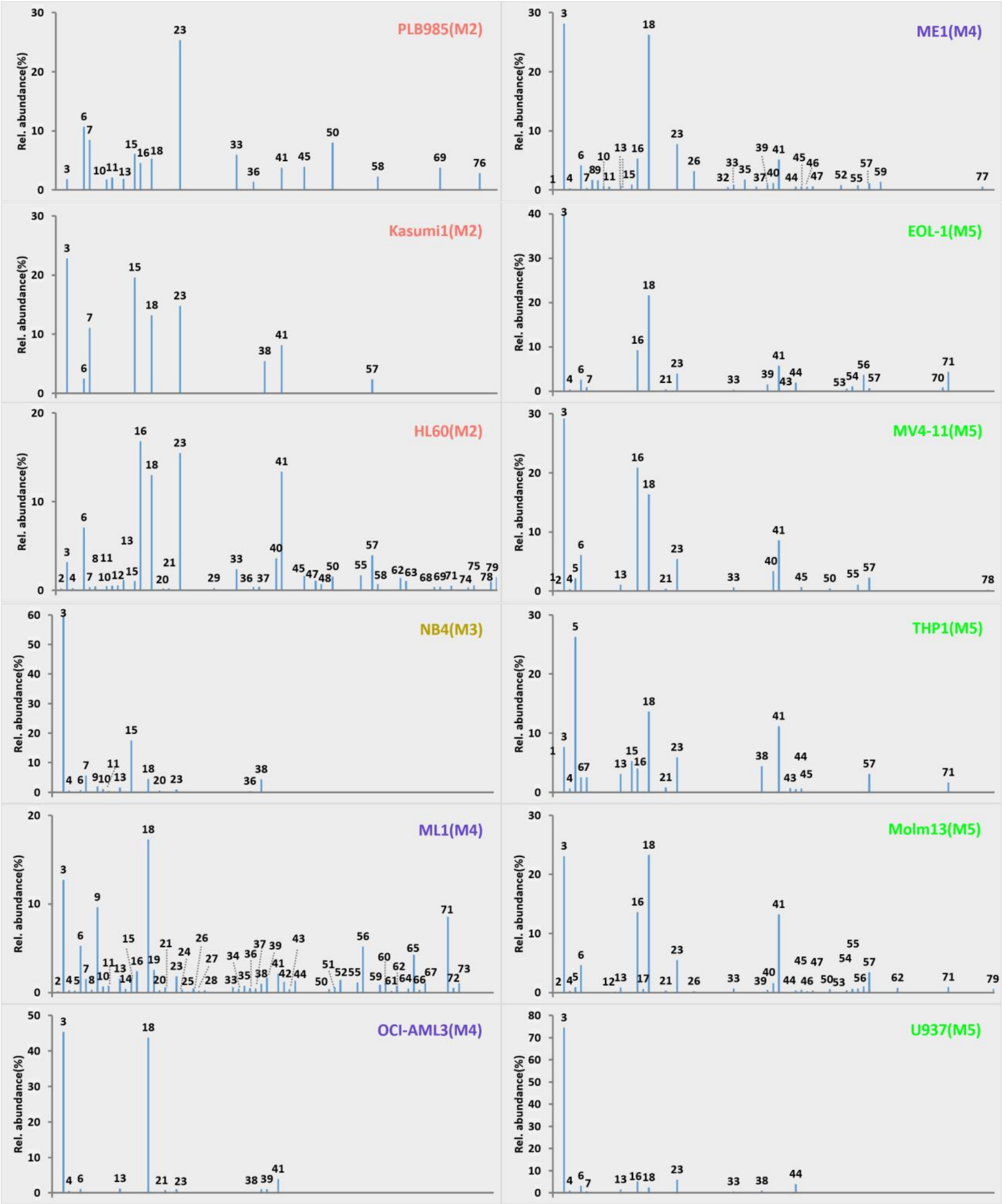

Supplementary Figure S-3. Structural diversity of GSL glycans in AML cell lines. Relative abundance of individual glycans derived from GSLs extracted from 19 AML cell lines. The glycans illustrated at the bottom of the figure (page 5) are the most abundant GSL glycans found in various AML cell lines (for a complete overview of the numbered glycans see **Supplementary Table S-2**). The reduced end is indicated by a circle at the reducing end of the glycans.

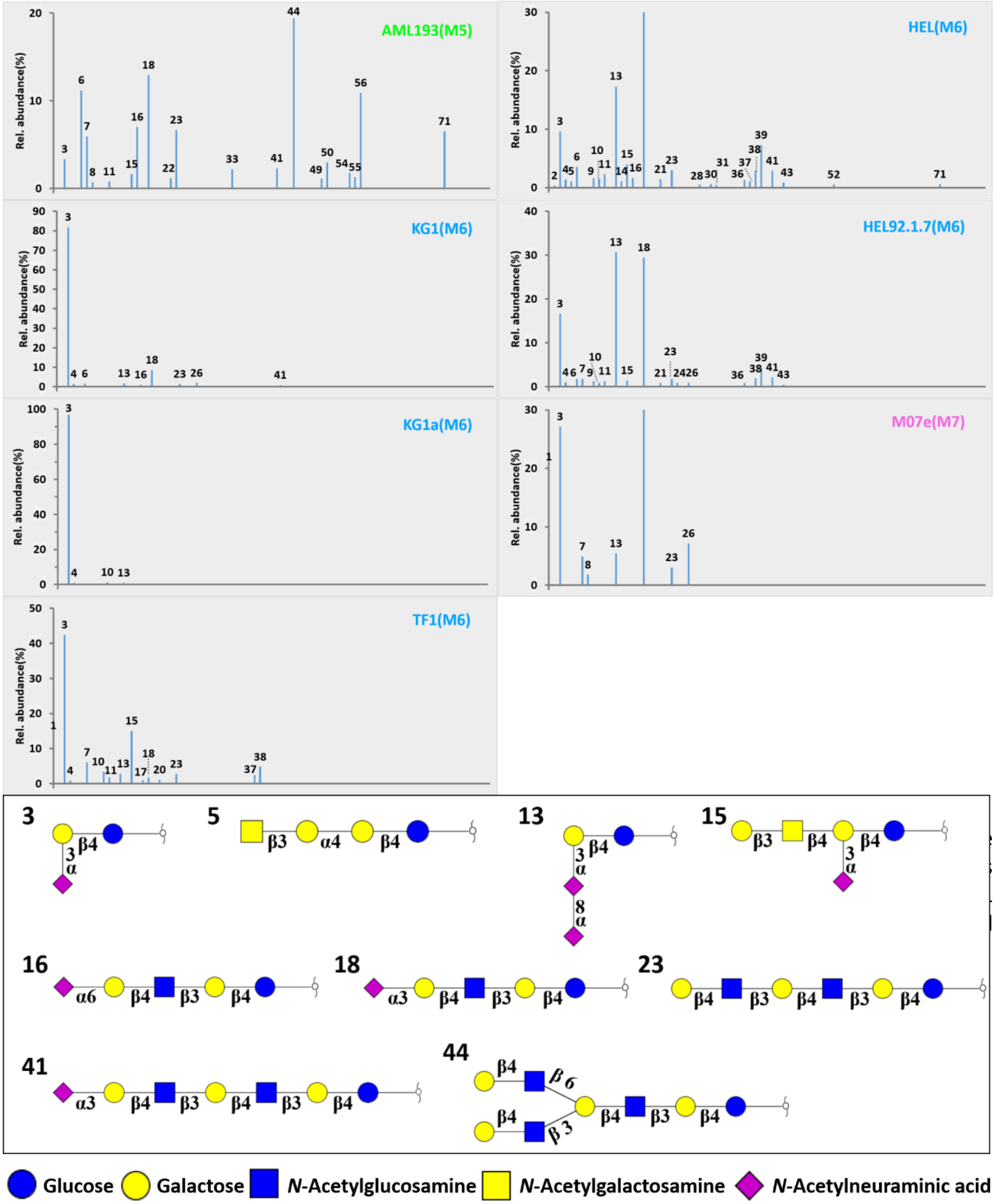
AML193(M5)
HEL(M6)
KG1(M6)
HEL92.1.7(M6)
KG1a(M6)
M07e(M7)
TF1(M6)
Rel. abundance(%)
3
5
13
15
16
18
23
41
44
Glucose
Galactose
N-Acetylglucosamine
N-Acetylgalactosamine
N-Acetylneuraminic acid

Figure S-4

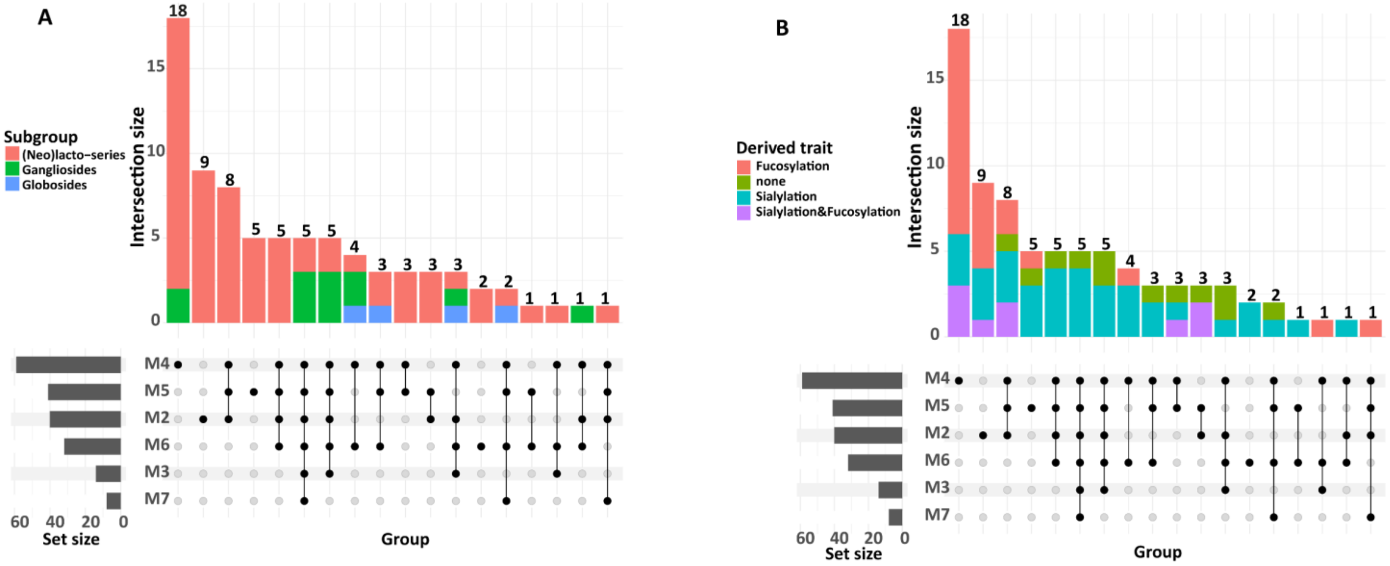

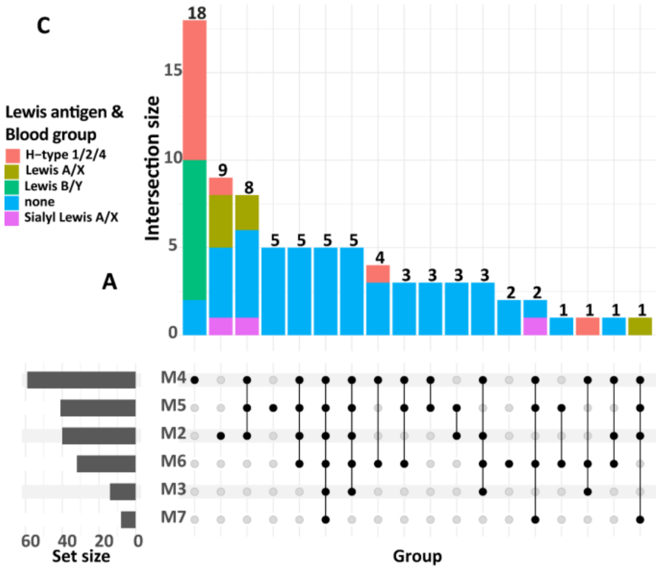

Supplementary Figure 4. The UpSet plots display the intersections between AML cell lines grouped by classes and (A) GSL glycan subgroups, (B) glycosylation traits, and (C) glycan antigens, respectively. (A) indicates how many glycans from each subgroup are specifically expressed in the respective AML classes. **(B)** displays how many fucosylated and/or sialylated glycans are specifically expressed in one or several different classifications of AML. **(C)** shows how many glycans with different antigens expressed in one or several different classifications of AML specifically. The bar chart on the left indicates the number of GSL glycans in each classification. The upper bar chart shows the intersection size between the different classifications (legend in the upper left corner). The dark circle indicates the sets that are part of the intersection.

Figure S-5

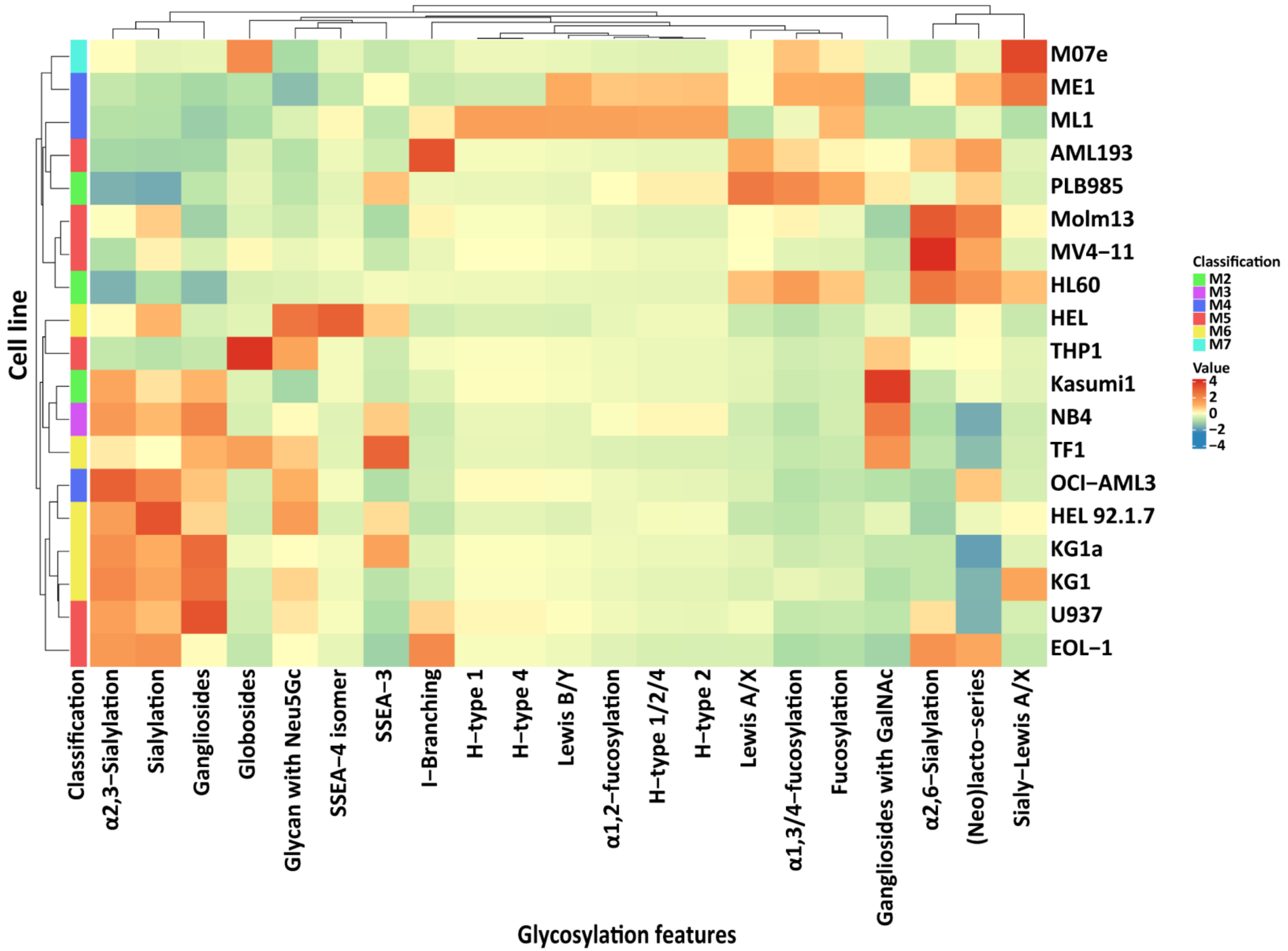

Supplementary Figure S-5. Distribution of GSL glycans features (derived traits) in specific AML cell lines illustrated in the heatmap. The relative quantification (%) of glycosylation features (bottom) for each cell line (right) was calculated. Different classifications of cell lines are indicated with color code based on FAB classification [1]. The standardization with mean equal to zero and standard deviation equal to one was performed.

Figure S-6

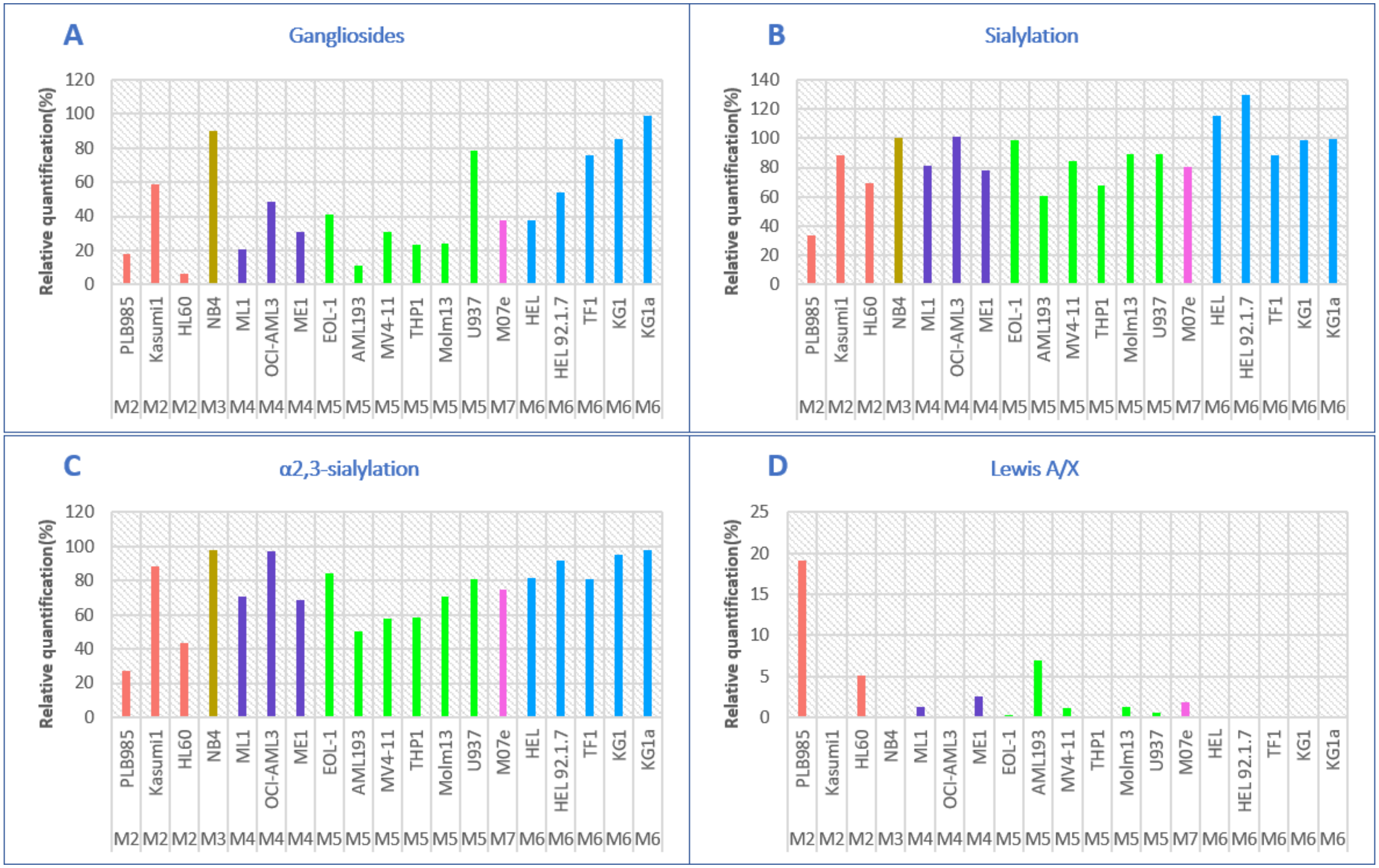

Supplementary Figure S-6. The distribution of glycosylation features in AML cell lines grouped by FAB classification. The expression of **(A)** Gangliosides, **(B)** Sialylation, **(C)** α2,3-sialylation and (**D**) Lewis A/X antigen in AML cell lines is displayed.

Figure S-7

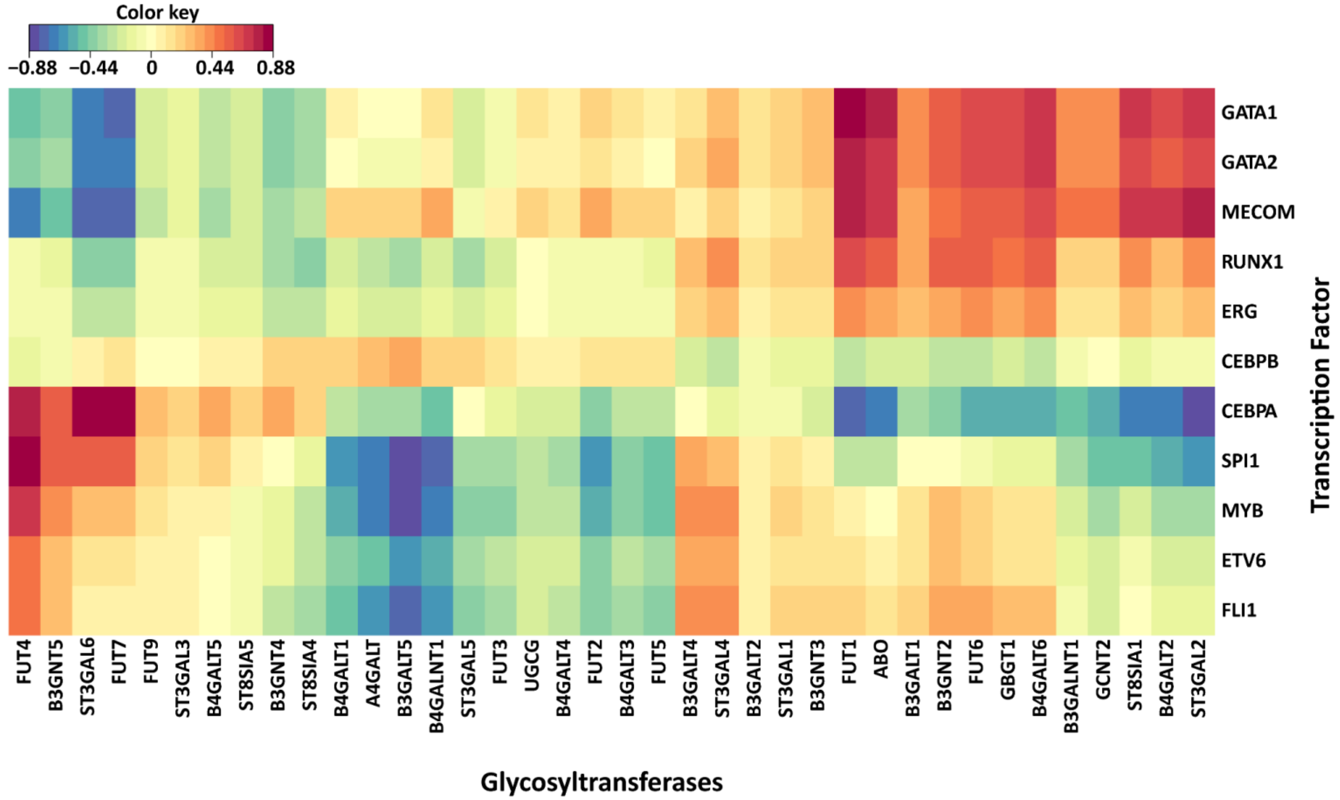

Supplementary Figure S-7. Canonical correlation analysis between gene expression of glycosyltransferases and hematopoietic transcription factors. The heatmap of canonical analysis indicates the correlation between glycosyltransferases (bottom) involving in the biosynthesis of GSL glycans and selected hematopoietic transcription factors (right). The dataset of gene expression of relevant GTs and TFs (bottom) was extracted from the Cancer Cell Line Encyclopedia. The degree of correlation is indicated in the top legend (blue: negative correlation; red: positive correlation).

Figure S-8

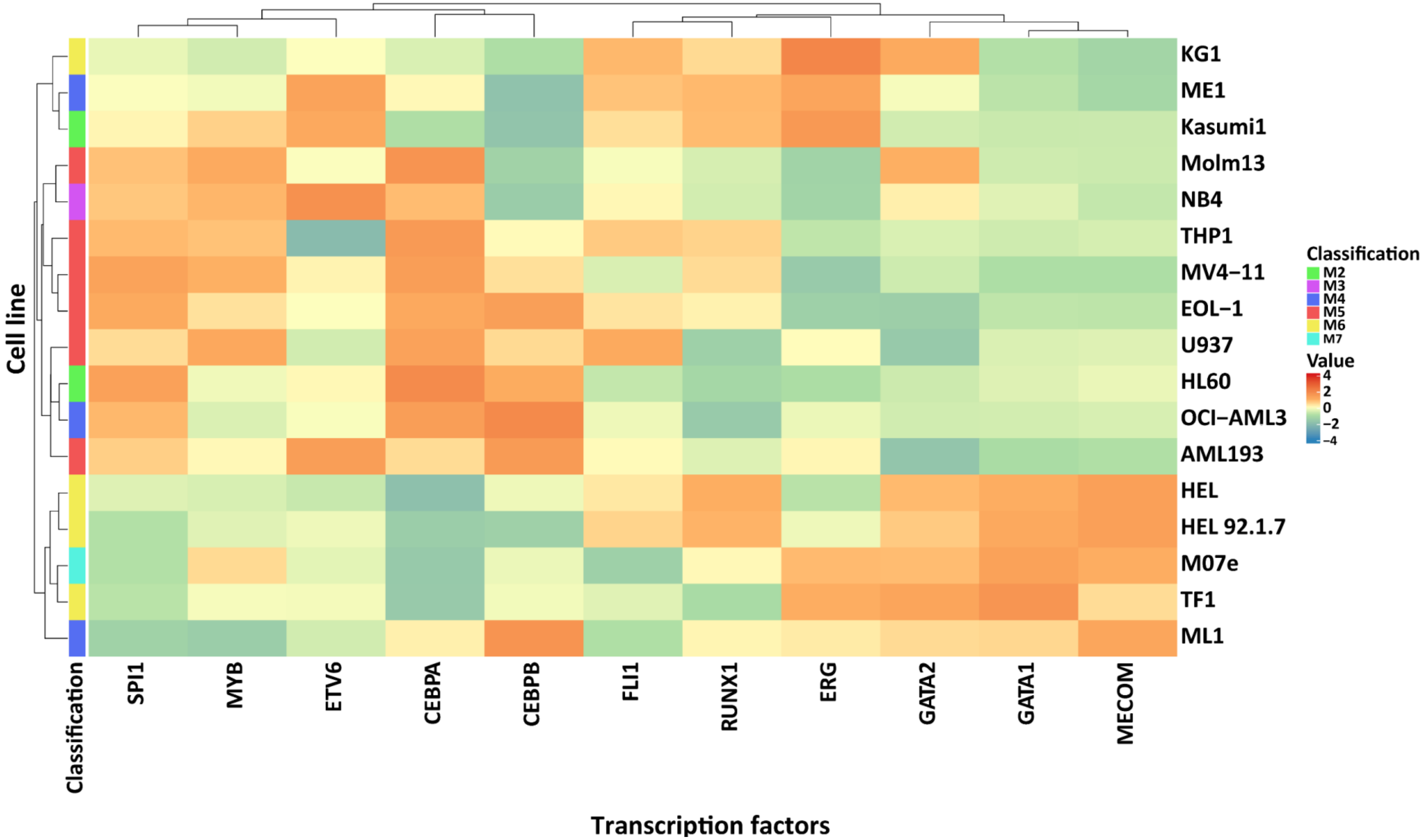

Supplementary Figure S-8. Distribution of gene expression of hematopoietic transcription factors in specific AML cell lines illustrated in the heatmap. The gene expression of hematopoietic transcription factors (bottom) for each cell line (right) was calculated. Different classifications of cell lines are indicated by a color code based on FAB classification. The dataset of gene expression of relevant TFs was extracted from the Cancer Cell Line Encyclopedia.

References

1. Quentmeier, H., Reinhardt, J., Zaborski, M. & Drexler, H.G. FLT3 mutations in acute myeloid leukemia cell lines. *Leukemia* **17**, 120-124 (2003).
